# Northeast African genomic variation shaped by the continuity of indigenous groups and Eurasian migrations

**DOI:** 10.1371/journal.pgen.1006976

**Published:** 2017-08-24

**Authors:** Nina Hollfelder, Carina M. Schlebusch, Torsten Günther, Hiba Babiker, Hisham Y. Hassan, Mattias Jakobsson

**Affiliations:** 1 Dept. of Organismal Biology, Uppsala University, Uppsala, Sweden; 2 Dept. of Linguistic and Cultural Evolution, Max Planck Institute for the Science of Human History, Jena, Germany; 3 Banoon ART and Cytogenetics Centre, Bahrain Defense Force Hospital, Manama, Kingdom of Bahrain; 4 SciLife Lab, Uppsala University, Uppsala, Sweden; University of Pennsylvania, UNITED STATES

## Abstract

Northeast Africa has a long history of human habitation, with fossil-finds from the earliest anatomically modern humans, and housing ancient civilizations. The region is also the gate-way out of Africa, as well as a portal for migration into Africa from Eurasia via the Middle East and the Arabian Peninsula. We investigate the population history of northeast Africa by genotyping ~3.9 million SNPs in 221 individuals from 18 populations sampled in Sudan and South Sudan and combine this data with published genome-wide data from surrounding areas. We find a strong genetic divide between the populations from the northeastern parts of the region (Nubians, central Arab populations, and the Beja) and populations towards the west and south (Nilotes, Darfur and Kordofan populations). This differentiation is mainly caused by a large Eurasian ancestry component of the northeast populations likely driven by migration of Middle Eastern groups followed by admixture that affected the local populations in a north-to-south succession of events. Genetic evidence points to an early admixture event in the Nubians, concurrent with historical contact between North Sudanese and Arab groups. We estimate the admixture in current-day Sudanese Arab populations to about 700 years ago, coinciding with the fall of Dongola in 1315/1316 AD, a wave of admixture that reached the Darfurian/Kordofanian populations some 400–200 years ago. In contrast to the northeastern populations, the current-day Nilotic populations from the south of the region display little or no admixture from Eurasian groups indicating long-term isolation and population continuity in these areas of northeast Africa.

## Author summary

Northeast Africa has geographic and historical links to Eurasia via the Middle East and the Arabian Peninsula, but the demographic history of the region itself has been more elusive. We investigate genomic diversity of northeast African populations and found a clear bimodal distribution of variation, correlated with geography, and likely driven by Eurasian admixture in the wake of migrations along the Nile. This admixture process largely coincides with the time of the Arab conquest, spreading in a southbound direction along the Nile and the Blue Nile. Nilotic populations occupying the region around the White Nile show long-term continuity, genetic isolation and genetic links to ancestral East African people. Compared to current times, groups that are ancestral to the current-day Nilotes likely inhabited a larger area of northeast Africa prior to the migration from the Middle East as their ancestry component can still be found in a large area. Our findings reveal the genetic history of Sudanese and South Sudanese people, broaden our knowledge on demographic history of humans, and quantify the impact of large-scale historic migration events in northeast Africa.

## Introduction

The Nile River Valley and northeast Africa have experienced a long history of human habitation. The region harbored some of the most ancient civilizations in the world and contains fossil finds of the earliest anatomically modern humans [1–3]. Agriculture has a long history in the Nile River valley, and crops of potential Near Eastern origin as well as sorghum found in Sudan have been dated to 3000BC [4]. Livestock was introduced into northeast African and Sudan in the 5^th^ millennium BC (likely from the North) and pastoralism spread rapidly across sedentary agriculturalists who lived along the Nile as well as to the nomadic populations inhabiting the drier surrounding regions [4]. Following the introduction of agriculture and pastoralism, settlements started growing, which led to the forming of political units. In Nubia (roughly the northern parts of current-day Sudan), the Kingdom of Kerma emerged around 3000 BC. Nubia has successively been at the center of several ensuing states, and the historical records show interactions with neighboring states through trade and confrontation, possibly reaching back to predynastic times [4–6]. Modern-day Sudan and South Sudan cover parts of the Nile River and the joining of the Blue and the White Nile, areas that link the northern part of the Nile Valley and North Africa with East Africa. Today, these areas display great linguistic diversity, with Sudan and South Sudan housing 137 living languages [7], which belong to three of the four linguistic macro-families found on the African continent: Afro-Asiatic, Nilo-Saharan, and Niger-Congo.

Previous genetic studies focusing on human history in Sudan and South Sudan have used uniparentally inherited markers [8–10], low density polymorphic autosomal markers [11–17], or were only covering a limited number of populations [18]. These studies have found substantial genetic differentiation in northeast Africa and indications of migration and admixture. For instance, Tishkoff, Reed [18] investigated more than one hundred African populations using some 800 microsatellites, including six populations from Sudan and South Sudan and showed that eastern Africa harbors substantial amounts of genetic diversity. However, wide ranges of populations, representative of all the main linguistic groupings, in and around Sudan and South Sudan have not been studied in order to decipher population history using high-resolution genome-wide data.

In this study we genotyped some 3.9 million SNPs in 221 individuals from a total of 18 populations from South Sudan and Sudan to investigate population structure and admixture patterns, which we use to reconstruct the genetic history of this region of northeast Africa. We find a genetic differentiation within the Sudanese and South Sudanese groups that is driven by Eurasian admixture, which may have followed the Nile southward and coincides with the time of the Arab conquest.

## Results/Discussion

**Fig 1:**
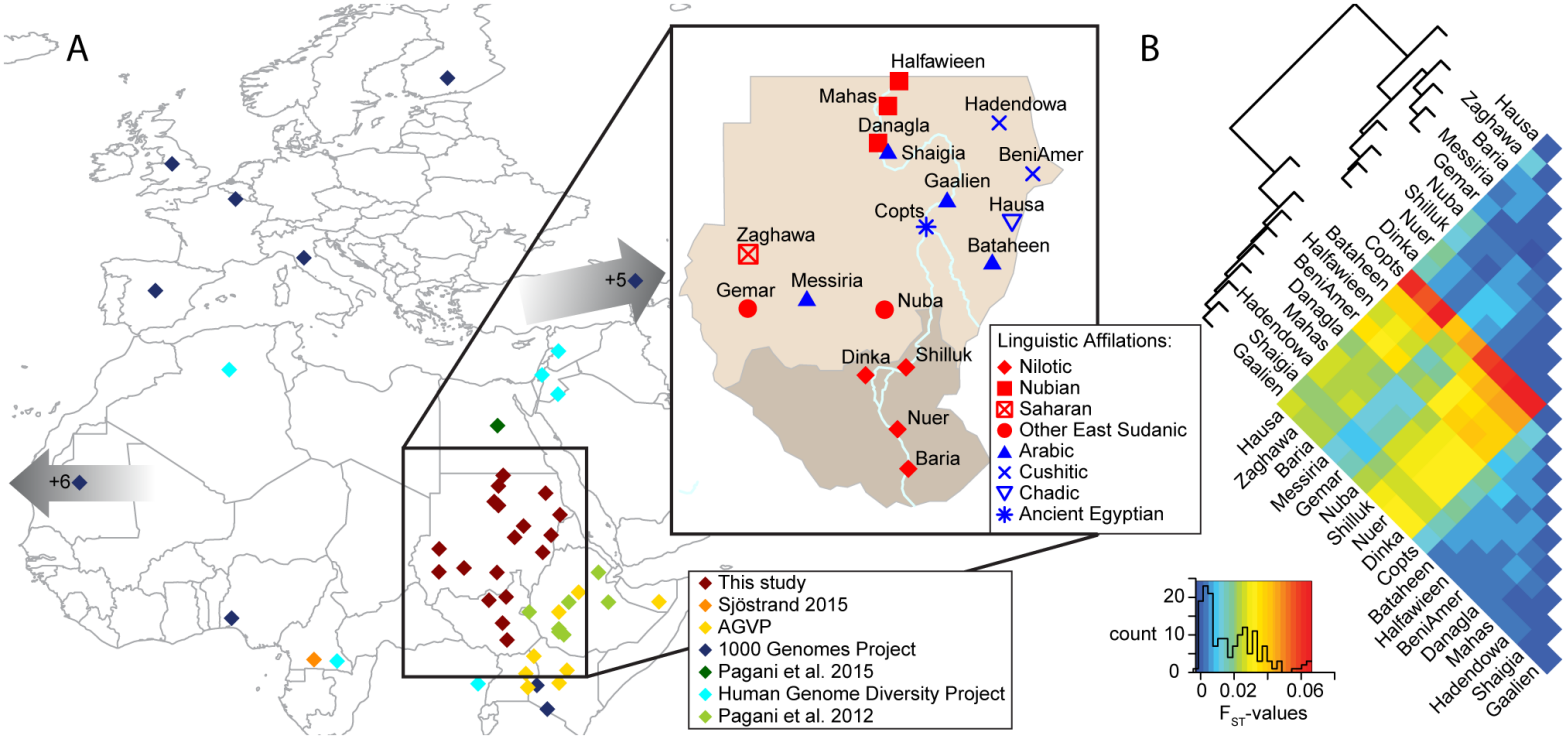
Overview of populations investigated in this study. (A) Partial map of Africa and Europe showing the populations investigated in this study. Gumuz and S.Sudanese were not included in the figure as the geographic sampling coordinates were unclear. Coordinates were approximated for the 1000Genome, HGDP, Egyptian, Nzime and Somali populations. This study includes eleven populations from the 1000Genome Project that have been sampled from areas outside of the map range, indicated by the arrows. Colors of the symbols indicate project affiliations. A zoom on Sudan and South Sudan shows the geographic midpoint of the populations sampled in this study. The colors of the symbols indicate linguistic affiliation, Nilo-Saharan speakers are shown in red and Afro-Asiatic speakers in blue. The Nuba, shown in red, also speak Kordofanian, a Niger-Congo language. (B) Pairwise F_ST_ of the Sudanese and South Sudanese populations. The key shows the F_ST_-values on the x-axis and the y-axis displays the amount of observed instances in a histogram. A UPGMA tree is shown that was calculated using the F_ST_ distance matrix.

We investigated the genetic variation of Sudanese and South Sudanese populations by genotyping 221 individuals sampled from 18 populations (Fig 1A, Table 1) using the Illumina Human Omni5MExome array. The sampled populations cover a range of languages belonging to three major linguistic families that include the sub-groupings; Semitic, Cushitic, Eastern Sudanic, Kordofanian, Ancient Egyptian, and Chadic (Fig 1A, Table 1). Some of the sampled populations have been suggested to be recent migrants to the area (such as the Hausa and Copts), while others are assumed to have a long standing history in Sudan (i.e. Nubians) and South Sudan (i.e. Nilotes) [11, 14, 18] (note that we will use population names and/or ethnic grouping, Table 1, when discussing the genetic results).

**Table 1:** Population names, sample sizes, ethnic and linguistic affiliations of the populations.

Population	Ethnicity	*n*	Linguistic Family	Linguistic subgroup
Bataheen	Arab	10	Afro-Asiatic	Semitic
Gaalien	Arab	14	Afro-Asiatic	Semitic
Shaigia	Arab	12	Afro-Asiatic	Semitic
Messiria	Arab	8	Afro-Asiatic	Semitic
BeniAmer	Beja	16	Afro-Asiatic	Cushitic
Hadendowa	Beja	11	Afro-Asiatic	Cushitic
Copts	Copts	14	Afro-Asiatic	Ancient Egyptian
Hausa	Hausa	5	Afro-Asiatic	Chadic
Nuba	Nuba	16	Nilo-Saharan and Niger-Congo	Eastern Sudanic and Kordofanian
Danagla	Nubian	15	Nilo-Saharan	Eastern Sudanic
Mahas	Nubian	15	Nilo-Saharan	Eastern Sudanic
Halfawieen	Nubian	11	Nilo-Saharan	Eastern Sudanic
Dinka	Nilotic	16	Nilo-Saharan	Eastern Sudanic
Nuer	Nilotic	15	Nilo-Saharan	Eastern Sudanic
Shilluk	Nilotic	16	Nilo-Saharan	Eastern Sudanic
Baria	Nilotic	5	Nilo-Saharan	Eastern Sudanic
Zaghawa	Zaghawa	15	Nilo-Saharan	Saharan
Gemar	Gemar	7	Nilo-Saharan	Saharan

Following quality filtering (~3.9 million SNPs remained, see SI), we merged the Sudan and South Sudan genotype dataset to relevant published genotype datasets from neighboring and other relevant populations [19–24] (Fig 1A, S1 Table) in order to bring the genetic variation into a regional and global context (SI, Method Section). This dataset is likely the most comprehensive dataset assembled to date of northeast African populations.

**Fig 2:**
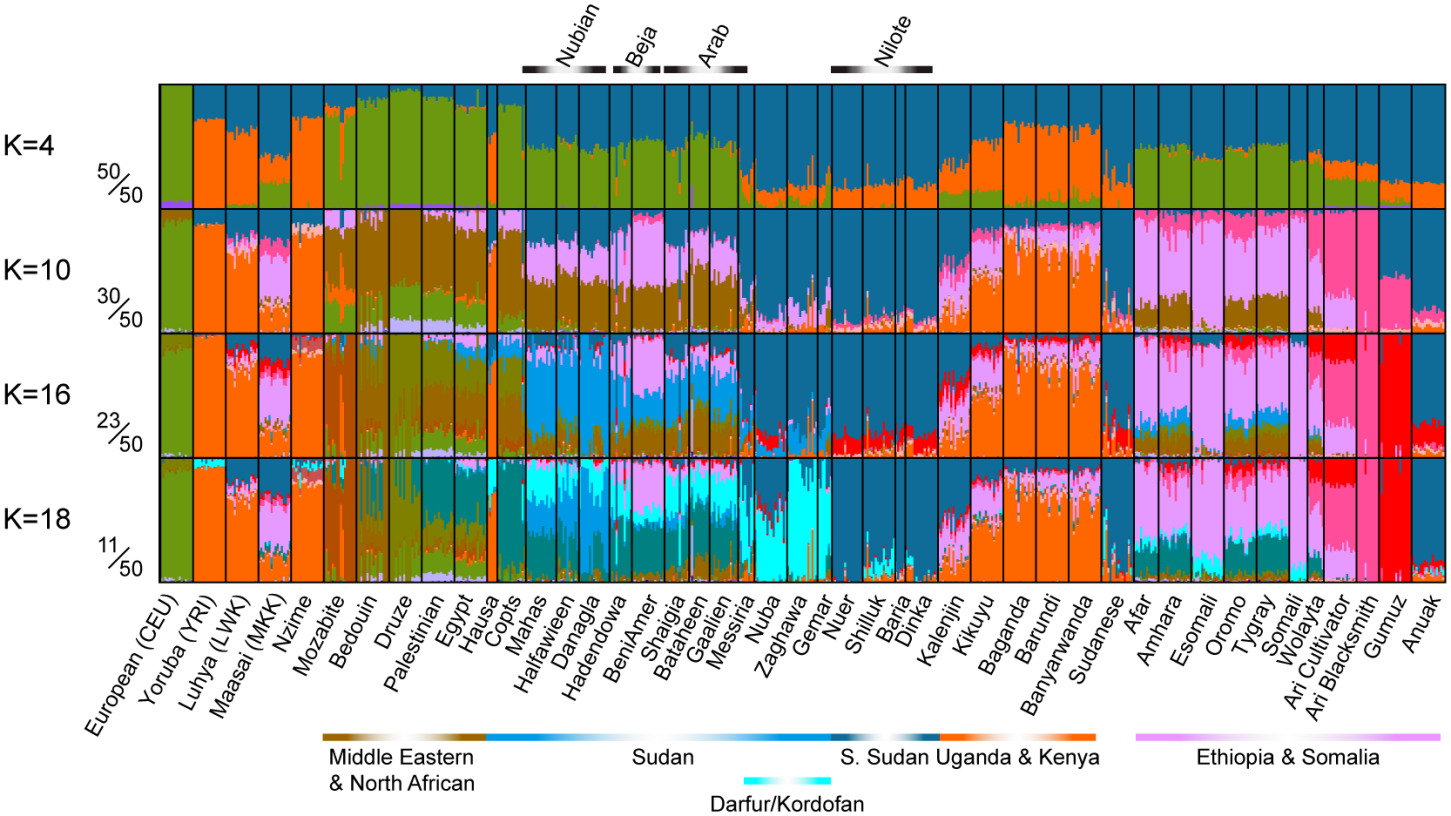
Inferred admixture fractions [51] for different choices of number of clusters. Four different numbers of clusters are displayed. The fractions on the left show the fraction of 50 replicate analyses that support of the most common and displayed mode [52]. Populations that did not contribute to the African variation were removed from this figure. See S3 Fig for the full range of populations and additional K. The bars on top represent the ethnic group, the bars on the bottom indicate geographic grouping.

Northeast African individuals and groups displayed marked levels of population structure and differentiation (Figs 1B and 2, S1–S6 Figs), and some groups showed strong affinities to groups from other areas, including Europe, Middle East and western Africa (Fig 2, S1–S6 Figs). Focusing on population structure in Sudan and South Sudan, we found that genetic variation was correlated with geography (r = 0.39, p<0.01, Mantel test), to a greater extent than to linguistic classification (r = 0.28, p<0.01), indicating that geography drives population structure in the area. Several populations, in particular from the North and East of Sudan displayed genetic affinities to non-Africans, which is consistent with recent admixture into these groups (Fig 2, S1–S6 Figs). This admixture unifies the Nubian, Arabic and Beja populations from the north, and it is almost completely absent in the western Sudanese and South Sudanese populations.

### Nilotic groups emerged from an ancestral group of East Africa

**Fig 3:**
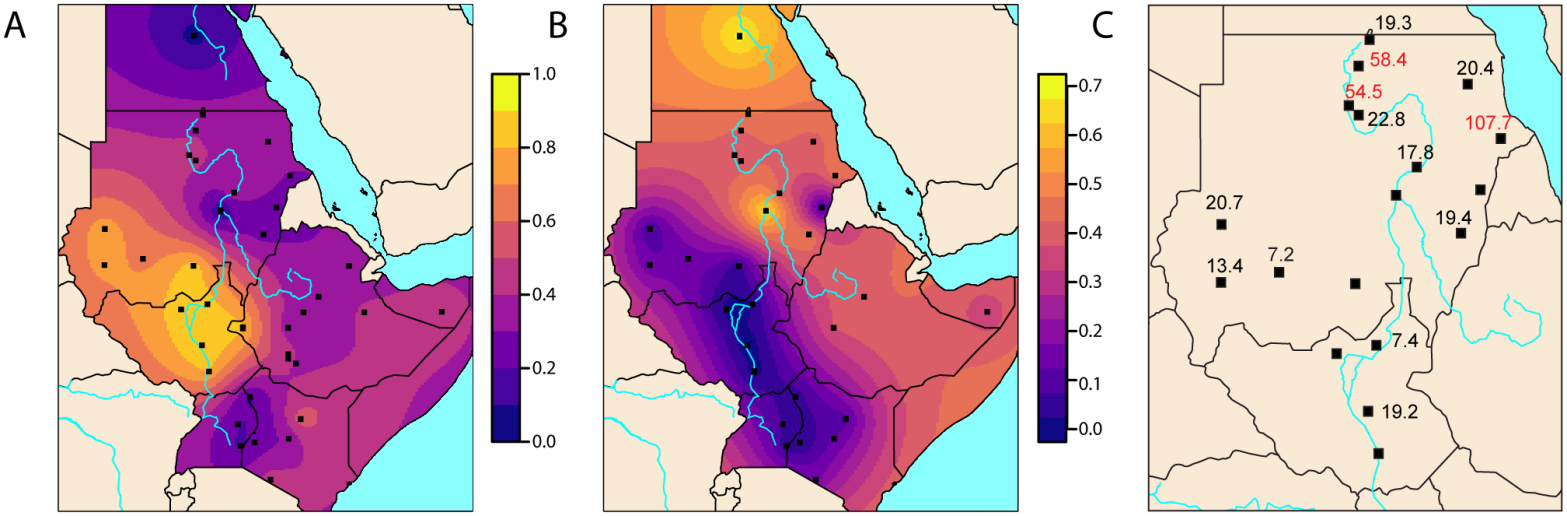
Maps showing the amount of Nilotic and Eurasian admixture and admixture dates in investigated populations. (A) Map shows the distribution of the Nilotic component in Northeast African populations (at K = 7). (B) Estimated non-African (using a European group) admixture using f_4_-ratios (see Methods). (C) Admixture dates (in generations) of Sudanese populations estimated using patterns of LD decay [34]. Numbers in red indicate multiple admixture events of which the oldest is shown. Populations without admixture dates had no significant results in the analyses for an admixture event between a Sudanese or South Sudanese population and a non-African population. See S7 Table for putative admixture sources.

Among the populations from Sudan and South Sudan, the four Nilotic populations formed a notable population cluster based on the genome-wide data. They were genetically uniform with little genetic differentiation among themselves (pairwise F_ST_ values ≤ 0.0028, Fig 1B, S7A Fig). In the ADMIXTURE analyses, the Nilotic populations retained a specific ancestry component (blue), which is shared with other northeast African groups at low values of K, where most of the Sudanese populations have a substantial fraction of this ancestry (Figs 2 and S1–S6). Even at higher values of K, the Nilotes formed their own ancestry component, a component found in modest proportions in populations from Sudan and South Sudan. The Nilotes also appeared as one of the most common source populations for other Sudanese and South Sudanese populations (Figs 2 and 3A). We furthermore compare the affinity between the Nilotes and Neolithic European farmers (represented by an individual from the Linearbandkeramik (LBK)), using the 4,500 year old Mota individual from Ethiopia to represent an East African group that has not been affected by Eurasian admixture in the last 4,500 years [25]. Testing the population tree D(Ju|’hoansi,LBK;Mota,Nilote) shows no support for an affinity between Neolithic European farmers and Nilotes (S8A Fig), as can also be seen from the f_4_-ratio estimates of Eurasian ancestry in Nilotes (Fig 3B, S9A Fig). Previous studies of uniparental or few markers also found little support for incoming gene-flow to the Nilotic populations [9, 11, 15, 25], and, taken together with our results, Nilotic populations appear to have remained relatively isolated over time.

The Nilotes are predominantly pastoralist populations, they live in Uganda, Ethiopia, Kenya, Tanzania, and are the most prominent ethnicity in South Sudan. They are traditionally strongly endogamic which could account for low levels of admixture. In terms of specific Nilotic populations, the f_3_ test showed no significant signal of gene flow with external populations for the Nuer and Baria (Fig 3A), however, we detected indications of external gene flow from West Africa (YRI) into Dinka (f_3_ = -0.001038, Z = -5.283) and TSI to Shilluk (f_3_ = -0.002565, Z = -7.951, S2 Table). These observations taken together, suggest long term isolation and continuity between the current-day Nilotic populations and the ancestral populations of northeast Africa.

### Little admixture in northeast Africa with Bantu-speaking groups

All the investigated Sudanese and South Sudanese populations, except the Hausa, showed almost no West African (orange in Fig 2) component or, at a higher K, Bantu component (Fig 2, yellow in S3 Fig) in the ADMIXTURE analysis. The Bantu migration that swept over most of sub-Saharan Africa 3–4 thousand years ago (kya) [26] did not cause massive admixture in northeast Africa, contrary to what has been found in many other sub-Saharan African regions, e.g. East Africa and southern Africa [18, 27, 28]. This expansion seems to have passed south of the Sudanese Nilotic populations in an eastward direction from West-Africa. The strongly endogamic Nilotic populations could have acted as a migration barrier for northeast Africa preventing admixture with Bantu-speaking groups of West African origin during the migrations of the Bantu expansion, potentially in addition to climatic barriers connected to the agriculture of the Bantu-speakers. Although there are a few Bantu speaking populations in South Sudan [29] that likely migrated during the Bantu expansion, they do not appear to have mixed much with local Nilotic groups.

The Afro-Asiatic speaking Hausa population from northeastern Sudan was the exception to the observation of little West African affinity in Sudan and South Sudan (Fig 1). The Hausa, originally of western Africa, comprises the largest West African population that have migrated to Sudan during the past 300 years, traditionally employed mainly in agricultural activities [30, 31]. In S11 Fig they cluster in between the West African Yoruba and Nzime, and the Darfurian/Kordofanian and Nilotic populations. This finding is consistent with previous analyses [18, 30, 32, 33]. Even though the ADMIXTURE analysis showed some level of local Nilotic genetic material (~30% at K11 and higher, Fig 2, S3 Fig), the f_3_ statistics did not provide significant evidence for admixture with Darfurian/Kordofanian and Nilotic populations. Using LD decay patterns [34], we estimate an admixture event in the Hausa to 31.2 ± 9.3 generations ago (Z = 3.34683) from a Eurasian source. This is before the historically documented settlement of the Hausa in the Sudan and it is still unknown if the Hausa populations of West Africa also show this admixture signal. These observations point to that the Hausa originated in West Africa and migrated recently to Sudan, where they have stayed relatively isolated from neighboring populations.

### Nubians are an admixed group with gene-flow from outside of Africa

**Fig 4:**
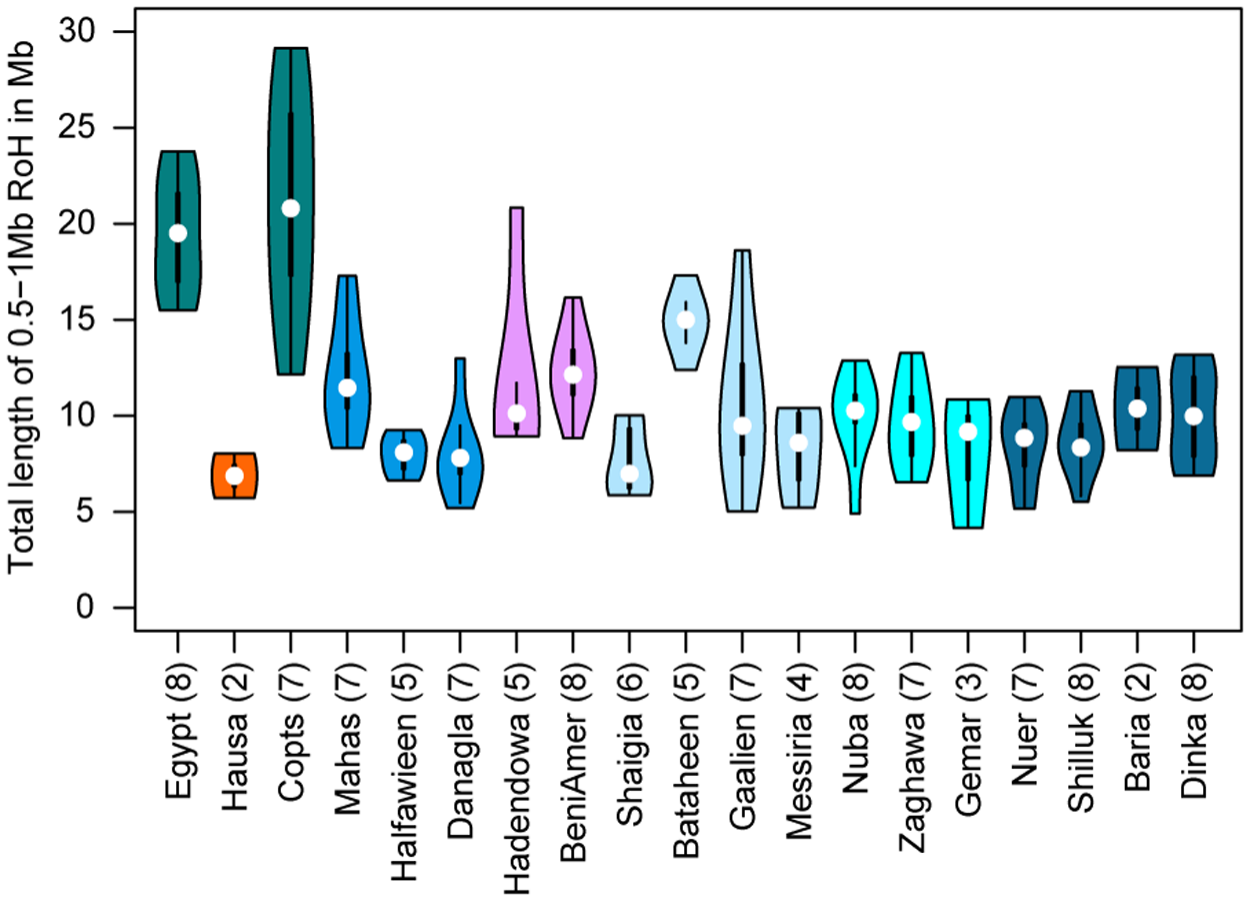
Distribution of total length of runs of homozygosity per individual per population. Runs of homozygosity are between 0.5–1 Megabases (Mb) in length. Runs of homozygosity were calculated on a chimeric dataset (SI).

The Nubians inhabit the Nile valley in the arid desert of northern Sudan and speak Eastern Sudanic languages of the Nilo-Saharan linguistic family that are close to the languages spoken by Nilotic populations (Table 1, Fig 1A). The Nubian populations have a long history in the region, dating back to dynastic Egypt [5]. They showed little genetic differentiation among individuals and groups, with a maximum (across all pairwise comparisons) pairwise F_ST_ (Weir and Cockerham’s estimator) of 0.004513 between the Mahas and the Halfawieen (Fig 1B, S7A Fig). The F_ST_ values to the surrounding Arabic and Beja populations were also low, which hints at gene-flow or shared ancestry with the neighboring populations. Even though the Nubians and the Nilotes are linguistically closer to each other than to the Afro-Asiatic groups, the Nubians showed the greatest genetic differentiation (F_ST_ between 0.02 and 0.04) to the Nilotes (Fig 1, S7A Fig). To investigate whether this signal of genetic differentiation is driven by the Eurasian admixture into the Nubians (as seen in Fig 2), we created pseudo-‘unadmixed’ (in terms of not having Eurasian admixture) allele frequencies (see SI) and calculated Wright’s F_ST_, which showed that an ‘unadmixed’ Nubian gene-pool is genetically similar to Nilotes (S7B Fig). The strongest signal of admixture into Nubian populations came from Eurasian populations (S10 Fig, S2 Table) and was likely quite extensive: 39.41%-47.73% (f_4_-ratio, Z-scores between 22.8 and 26.7 Fig 3B, S9 Fig). Interestingly, the Nubians showed the highest level of allelic richness, number of private alleles and shared private alleles (ADZE, between Danagla and Halfawieen, S12 Fig) among all Sudanese and South Sudanese groups. This observation together with a smaller total length of runs of homozygosity, between lengths of 0.5–1 kilobases, points to substantial admixture in Nubians (Fig 4). Hence, the Nubians can be seen as a group with substantial genetic material relating to Nilotes that later have received much gene-flow from Eurasians (likely Middle Eastern) and from East Africans (Fig 2).

### West-Eurasian migration from the north

All the populations that inhabit the Northeast of Sudan today, including the Nubian, Arab, and Beja groups showed admixture with Eurasian sources and the admixture fractions were very similar. The admixture component in the northeastern groups cluster with the greater European and Middle Eastern group assuming few clusters, and for greater number of assumed clusters, when a predominantly Middle Eastern cluster emerged, the admixture in northeastern Sudan connected to the Middle East (ADMIXTURE, Fig 2, f_3_, S10 Fig). According to historical and linguistic studies, and recent Y-chromosome data it has been suggested that the northeastern Sudanese populations especially Nubians and Beja were strongly affected by Eurasian migrations since the introduction of Islam from the Arabian Peninsula through Egypt and the Red Sea starting around 651 A.D [9, 35].

Assuming that the Nubian population is a mixture of an incoming Eurasian (TSI is used as a proxy) group and a resident group that is genetically similar to the current day Nilotes (Nuer is used as a proxy), first contact is dated using patterns of LD-decay [34] to roughly 56 generations ago for the Danagla (54.45 ± 10.34, Z = 5.26437) and the Mahas (58.35 ± 12.2, Z = 4.78402); the Halfawieen have received Eurasian admixture later, around 19 generations ago (19.31 ± 3.81, Z = 5.05949, S7 Table, Fig 3C). Assuming a generation time of 30 years, the admixture dates for Danagla and Mahas predate the Arab expansion in the 7^th^ century, and may suggest that the migrations and admixture predate Islamic conquest. However, the confidence intervals overlap with the 7^th^ century, and these admixture estimates largely coincide with the Arab expansion into the northeast of Sudan. It is known from historic sources that Arabic groups encountered the Nubians first in the 7^th^ century, and were held back from advancing further into the Sahel until the fall of Dongola in 1315/1316AD [36] and the collapse of the Kingdom of Makuria. This is consistent with the later date for the admixture into Halfawieen and the Arabic populations of Sudan. Previous studies [37, 38] have found a similar pattern for populations of Maghreb, where admixture times coincide with the time of the historically documented Arab conquest.

The Eurasian migrations also appear to have expanded and migrated into northeast Africa where they admixed with local populations giving rise to Arabic-speaking groups (Shaigia, Gaalien and Bataheen) that today inhabit areas of central Sudan (Fig 2). We further tested the source of admixture into the central Sudan Semitic speaking Arab groups (Shaigia, Gaalien and Bataheen) using ancient samples from Europe (LBK) and East Africa (Mota) and the population history of D(Ju|’hoansi,LBK;Mota,X), (where the Ju|’hoansi is an outgroup Khoe-San population from Namibia), which suggested Eurasian admixture into central Sudan Arab groups (see SI, S8A Fig). This migration and admixture occurred later than the events that brought Eurasian gene-flow into the Nubians (S3 Table, Fig 3C). Interestingly, when we overlay the Eurasian genetic component onto a geographic map, it appears as if the expansion could have spread along the Blue Nile (Fig 3B and 3C), showing a gradient of higher to lower admixture proportion and older to younger admixture dates from northern Sudan to South Sudan. The Eurasian admixture proportion in the Arab populations is high, ranging between ~40%–48% (SI, Fig 3B and S9A Fig). The presence of a northeast African genetic signature similar to Nilotic populations and the recent admixture signal from Eurasia indicates that the populations in central Sudan that self-identify as Arab were originally a local northeast African population (similar to the Nubians and the Beja) that mixed with a Eurasian population during the Arab expansion, or possibly earlier. However, the mixed groups kept the language and culture of the incoming migrants.

Beja groups, who generally reside in eastern areas of Sudan close to the sea, show high non-African admixture in all tests (Figs 2 and 3B, S1–S6 and S8–S10 Figs). The Beni Amer also showed a strong admixture signal with a Eurasian population as well as a shared ancestry component with the Somali population (pink component in Fig 2), which suggest admixture with the East African Cushitic-speaking populations, perhaps as a result of migration along the coast. We dated the admixture of the Beja populations with the Cushitic-speaking Somalian population [39], and the admixture dates go far back in time, about 59 generations ago for the Hadendowa and about 68–75 generations for the Beni Amer (S3 and S4 Tables). The large proportion of the East African (pink in Fig 2) component is therefore not a result of recent admixture of East Africans into the Beni Amer. Admixture of non-Africans into the Beni Amer was also dated to an early event about 107.7 ± 24.4 generations ago (Z = 4.41711) and a younger event, 34.2 generations ago (± 9.6, Z-score = 3.55532 Fig 3C, S7 Table) suggesting an early migration from Eurasian into these coastal African populations, possibly across the sea. However, these old admixture events into the Beni Amer could be driven by admixture from the Cushitic-speaking populations of the Horn of Africa, which themselves have received 30–50% non-African ancestry about 100 generations ago, or 3kya [22, 40].

### The population history of the Copts and their relation to Egyptians

The Copts represent a well-known ethnic group, generally practicing Christianity, which migrated from Egypt to Sudan around 200 years ago, settling in a predominately Muslim region. The ADMIXTURE analyses and the PCA displayed the genetic affinity of the Copts to the Egyptian population (Fig 2, S1–S6, S11 and S13–S16 Figs). Assuming few clusters, the Copts appeared admixed between Near Eastern/European populations and northeastern Sudanese and look similar in their genetic profile to the Egyptians. Assuming greater number of clusters (K≥18), the Copts formed their own separate ancestry component that was shared with Egyptians but can also be found in Arab populations (Fig 2). This behavior in the admixture analyses is consistent with shared ancestry between Copts and Egyptians and/or additional genetic drift in the Copts [41, 42].

The Copts and the Egyptians have a historically documented shared history. We further investigate the relationships of the Copts and the Egyptians to other groups. All population histories tested in every possible combination of either Copts or Egyptians, and Bedouin and Nuer, with Ju|’hoansi as outgroup to the others were rejected (D-statistic, |Z|>5.5), which points to a non-tree-like history of the Copts and Egyptians. Our results instead indicate that they are an admixed population of at least one sub-Saharan population and one Eurasian population, but had subsequent admixture with additional groups. The population tree that has the most support finds the Nuer (Nilotic) as an outgroup to the Bedouin and Copts (D(Ju|’hoansi,Nuer;Bedouin,Copts) = 0.0103, Z = 5.550). The Copts were estimated to be of 69.54% ± 2.57 European ancestry and the Egyptians of 70.65% ± 2.47 European ancestry (f_4_-ratio, Fig 3B, S9A Fig).

The Egyptians and Copts showed low levels of genetic differentiation (F_ST_ = 0.00236, Fig 1B), lower levels of genetic diversity (S17 Fig) and greater levels of RoH (Fig 4) compared to other northeast African groups, including Arab and Middle Eastern groups that share ancestry with the Copts and Egyptians (Fig 2) [41]. A formal test (D(Ju|’hoansi,X;Egypt,Copt)), did not find significant admixture into the Egyptians from other tested groups (X) as the explanation of the (admittedly low level of) differentiation between the two groups, and the Copts and Egyptians displayed similar levels of European or Middle Eastern ancestry (S8A and S8B Fig). Taken together, these results point to that the Copts and the Egyptians have a common history linked to smaller population sizes, and that the Copts have remained relatively isolated since the arrival to Sudan with only low levels of admixture with local northeastern Sudanese groups (S8B Fig).

### Populations of Darfur and Kordofan

The Messiria, a Semitic speaking Arab population, are nomads who inhabit a wide area in the Darfur and Kordofan regions. They were genetically closer to other Darfurian/Kordofanian populations than to the Arab populations of central Sudan (Fig 2, S3 Fig). The Messiria were clearly genetically differentiated from the Arab populations of northeastern Sudan (F_ST_ values of 0.0083–0.0229, compared to 0.0–0.0056 to Darfurian/Kordofanian populations, Fig 1B) while the other Arab populations of central Sudan were genetically closer to each other (F_ST_ 0–0.0052, Fig 1B). The Messiria showed a significant signal of admixture between Nilotes (Nuer) and Eurasians (TSI), but the signal was stronger for other Arabs (S8 and S10 Figs). The Eurasian fraction in the Messiria was about 15% compared to the (40%-48%) in the northeastern Arabic populations (Fig 3B). The admixture was dated to about 7 generations ago (S3 Table, Fig 3C). This points to the Messiria being a local Kordofanian population that has acquired the language and culture from an incoming Semitic population that they mixed with some 200 years ago (190–244 years ago assuming a generation time of 30 years, Z = 3.19695).

The Gemar, a Nilo-Saharan speaking population of Darfur and Kordofan also showed signals of Eurasian admixture (f_3_, S10 Fig) estimated to ~13% (Fig 3B, S9A Fig). This admixture event was dated at 13.36 ± 2.99 generations ago (Malder, S7 Table, Fig 3C). However, a proposed population tree of LBK as an outgroup to Mota and Gemar was supported (S8 Fig), suggesting that the Gemar traces much of their ancestry back to ancestral groups of east Africa. The Zaghawa and the Nuba showed very little Eurasian admixture (Figs 1, 2, S8 and S10) and they showed low genetic differentiation to the Gemar and the Messiria as well as to the Nilotic populations suggesting common ancestry of Nilotic, Darfurian and Kordofanian populations (Figs 1B and 2, S7 Fig).

## Conclusion

We have shown that there has been long-term migration into Sudan, moving in a southward direction possibly along the Nile and the Blue Nile. From historic documents, we know that the ancient Egyptians were in contact with the ancient Nubians that inhabited the Nile area in the north of modern-day Sudan. Our study suggests that the later migration followed along the Nile, likely being held up by the Nubians until the fall of the Kingdom of Makuria in the 14^th^ Century [4]. Following that historic event, the Arab expansion spread further southward, which can be seen in a succession of admixture events that occur more recent in time as one travels south. Many populations in Sudan that self-identity as Arab, displayed a population history of local Sudanese populations that have admixed with incoming Eurasian populations, and adopted the language and culture of the incoming migrants. In fact most populations from northeast Sudan (Nubian, Arab and Beja groups) seem to be a mixture of Middle Eastern and local northeast African genetic components, although only the Arab groups shifted to the Semitic languages. Cultural and linguistic replacement following the Arab conquest has been described previously in populations of the Maghreb [37, 38, 43].

The Eurasian admixture had less impact on the populations of western Sudan and South Sudan. The Darfurian and Kordofanian populations showed overall less admixture from non-African groups than the northeastern populations (and the limited admixture that does exist is more recent in time). The Nilotic populations have stayed largely un-admixed, which appears to be the case in Ethiopia too, where a similar observation has been made for the Gumuz [23, 44], an Ethiopian Nilotic population that is genetically similar to South Sudan Nilotes. Northeast African Nilotes showed some distinction from an ancient Ethiopian individual (Mota, found in the Mota Cave in the southern Ethiopian highlands), which suggests population structure between northeast and eastern Africa already 4,500 years ago. The modern-day Nilotic groups are likely direct descendants of past populations living in northeast Africa many thousands of years ago.

## Methods

### Preparation of samples

The DNA samples were chosen from a set of individuals that had been typed with 15 forensic microsatellites [11]. Blood samples were collected by Dr. H. Babiker with a permission from the Forensic DNA lab in Khartoum, Sudan, in 2009. The research purpose of population genomic investigations was described to each participant, and an informed written and oral consent was obtained from all participants. The samples were prepared for analysis using Whatman FTA Protocol BD09 and slightly adjusted Whatman FTA Protocol BD01 (SI). The samples were amplified using Illustra Genomiphi V2 DNA Amplification Kit following the protocol from Pinard, de Winter [45]. Genotyping was performed on an Illumina Human Omni5MExome SNP-array. Data filtering was performed using PLINK v1.07 and custom scripts (S18 and S19 Figs).

Datasets of different sizes were created to include neighboring and other relevant populations, weighing the amount of SNPs against the number of reference populations. Dataset 1 contains the novel populations and the Nzime [24] (~3.5 Million SNPs), dataset 2 contains the populations of dataset 1 and populations from [19, 20, 23] (1.4 Million SNPs), and dataset 3 containing dataset 2 and populations from [22, 46] (~220 thousand SNPS) (S17 Fig). Due to the risk of allelic drop-out (for some individuals) caused by imperfect whole genome amplification, which can result in the appearance of hemizygous stretches (SI), we also created a ‘haploidized’ dataset by randomly picking one allele at each position (if variable). This ‘haploidized’ dataset will avoid underestimating diversity in population samples even in the presence of some level of allelic drop-out (SI-Summary statistics). All results performed on diploid datasets were verified by repeating the analyses with the ‘haploidized datasets’ (S1–S6, S13–S17 and S20–S22 Figs). The datasets were furthermore merged with the Ju|’hoansi population from Namibia (to act as an outgroup), and two ancient individuals, an ancient Ethiopian (Mota), to provide an African sample with no European admixture [25], and a European Linearbandkeramik individual (LBK) as a European reference of Neolithic times [47] (S9, S23 and S24 Figs).

### Population genetic analyses

We computed genetic diversity within populations (Heterozygosity, runs of homozygosity) and between populations (Weir and Cockerham’s estimator of F_ST_, Wright’s F_ST_), using plink v1.07, v1.9 [48, 49] and in-house scripts. A Mantel test was performed to calculate the correlation of genetic to linguistic and geographic distances (S25 Fig). Measurements of allelic richness, number of private alleles and uniquely shared alleles were computed using ADZE [50] on allelic and haplotype-based data. S27 Fig shows that the pattern is not driven by ascertainment bias.

Patterns of population structure was investigated using ADMIXTURE [51], CLUMPP (v. 1.1.2, [52] and distruct v. 1.1 [53]. Formal tests of admixture (f_3_ test, D-statistic) were performed using admixtools [39]. f_3_(Nuer,TSI;X) was used to estimate non-African admixture and f_3_(X,Mota;Ju|’hoansi) was used to estimate ancestral East African affinity. D-statistics were calculated as D(Ju|’hoansi,LBK; Mota, X).

The time in generations of admixture was calculated using a haploidized version of the data (see SI) with Malder [34] and Rolloff [39] and converted to calendar years assuming 30 years/generation. An ancient individual has shown widespread back admixture into East Africa [25] from Eurasia. To formally quantify the extend of the Eurasian admixture proportion we performed f_4_-ratios on dat2a, calculated as f_4_(CHB,GBR;X,LBK)/f_4_(CHB,GBR;Mota,LBK) similar to Gallego Llorente, Jones [25]. The ancient Ethiopian (Mota) [25] was used as an ancestral unadmixed (in terms of no Eurasian admixture) East African sample and the LBK individual [47] to substitute for an ancient Eurasian population.

## Supporting information

S1 Text Detailed methods and additional results. (PDF)

S1 Fig Inferred admixture fractions using ADMIXTURE [51] for diploid dat1 for the clusters 2–7. The cluster number can be found on the left along with the amount of iterations that support this cluster out of 50 (CLUMPP) [52, 53].
(PDF)

S2 Fig Inferred admixture fractions using ADMIXTURE [51] for diploid dat2 for the clusters 2–15. The cluster number can be found on the left along with the amount of iterations that support this cluster out of 50 (CLUMPP) [52, 53].
(PDF)

S3 Fig Inferred admixture fractions using ADMIXTURE [51] for diploid dat3 for the clusters 2–20. The cluster number can be found on the left along with the amount of iterations that support this cluster out of 50 (CLUMPP) [52, 53].
(PDF)

S4 Fig Inferred admixture fractions using ADMIXTURE [51] for dat1h for the clusters 2–7. The cluster number can be found on the left along with the amount of iterations that support this cluster out of 50 (CLUMPP) [52, 53].
(PDF)

S5 Fig Inferred admixture fractions using ADMIXTURE [51] for dat2h for the clusters 2–15. The cluster number can be found on the left along with the amount of iterations that support this cluster out of 50 (CLUMPP) [52, 53].
(PDF)

S6 Fig Inferred admixture fractions using ADMIXTURE [51] for dat3h for the clusters 2–20. The cluster number can be found on the left along with the amount of iterations that support this cluster out of 50 (CLUMPP) [52, 53].
(PDF)

S7 Fig Fst. (A) Weir and Cockerhams estimator of FST. (B) Wrights FST for pseudo-unadmixed allele frequencies.
(PDF)

S8 Fig D-Statistic results. (A) Results for D(Ju|’hoansi, LBK; Mota, X) to account for non-African admixture in population X, where X is the population on the y-axis. (B) Results for D(Ju|’hoansi, X; Egypt, Copt) to investigate whether Egyptians or Copts received more admixture of source X, where X is the population on the y-axis.
(PDF)

S9 Fig f4 ratios. (A) shows the Mota-like proportion in the populations on the Y axis. Horizontal bars display 2SE. (B) shows the Neanderthal-like proportion in the populations on the Y axis. Horizontal bars display 2SE. (C) Correlation of the Neanderthal and European proportions (r = 0.925). The European proportion was calculated as 1- Mota-like proportion.
(PDF)

S10 Fig f3 results using Nuer and TSI as sources. On the Y axis are the target populations. The lines around the circle show 2SE.
(PDF)

S11 Fig PCA dat3a. PC1 describes the variation between Africa—non-Africa. PC3 describes the African variation and differentiates the Pygmies, West Africans and East Africans.
(PDF)

S12 Fig (A) Allelic richness and (B) private allelic richness for the Sudanese and the South Sudanese populations computed on a non-merged dataset using ADZE [5]. Shared Alleles (C) between populations within an ethnic group are highlighted. All possible pairwise combinations are shown in gray. The highest amount of shared alleles is found between the Danagla and Halfawieen.
(PDF)

S13 Fig Principal component analysis for PC1 –PC4 in dat1. (A) No outlier removal. (B) Five outliers removed.
(PDF)

S14 Fig Principal component analysis for PC1 –PC4 in dat1h. (PDF)

S15 Fig Principal component analysis for PC1 –PC4 in dat2. No outlier removal. (A) Diploid dataset. (B) Haploid dataset.
(PDF)

S16 Fig Principal component analysis for PC1 –PC4 in dat3. No outlier removal. (A) Diploid dataset. (B) Haploid dataset.
(PDF)

S17 Fig Heterozygosity plot. Heterozygosity is shown on the Y-axis. Sudanese populations are colored according to linguistic affiliation. Orange = Chadic, teal = Ancient Egyptian, blue = Nubian/Eastern Sudanic, pink = Cushitic, brown = Semitic, cyan = various Eastern Sudanic, and dark blue = Nilotic/Eastern Sudanic. (A) shows the heterozygosity for dat3. (B) shows the heterozygosity after the dataset was haploidized and chimeric individuals were created. This decreases the sample size by more than 50 percent.
(PDF)

S18 Fig Comparison of heterozygosity between datasets of different amount of SNPs to see the effect of ascertainment bias. Average value of heterozygosity per population after removal of one outlier in the Nzime in dat1.
(PDF)

S19 Fig Illustration of platform bias. Samples are colored according to genotyping platform they were genotyped on. Population labels are displayed on the median of the individual values.
(PDF)

S20 Fig Distribution of runs of homozygosity for the Sudanese populations of the unmerged phased dataset. The average total length of the genome in runs of homozygosity in a number of length categories is plotted for each Sudanese population. Error bars represent one standard deviation. (A) Runs of homozygosity for the diploid dataset. (B) Runs of homozygosity of the chimeric unmerged dataset. Legend applies to both plots.
(PDF)

S21 Fig Distribution of runs of homozygosity for dat2. The average total length of the genome in runs of homozygosity in a number of length categories is plotted for each Sudanese population. Error bars represent one standard deviation. (A) Runs of homozygosity for the diploid dataset. (B) Runs of homozygosity of the chimeric unmerged dataset. Legend applies to both plots.
(PDF)

S22 Fig Comparisons of total length of runs of homozygosity and number of runs of homozygosity per individual for dat1. (A) Diploid dataset.(B) Chimeric dataset.
(PDF)

S23 Fig Outgroup f_3_. Measured shared drift of the populations on the Y axis with ancient Ethiopian individual. Lines indicate 2SE.
(PDF)

S24 Fig Inferred admixture fractions using ADMIXTURE [51] for dat3a for the clusters 2–15. The cluster number can be found on the left along with the amount of iterations that support this cluster out of 50 (CLUMPP) [52, 53]. The ancient individuals are on the right.
(PDF)

S25 Fig Linguistic distances. Classification according to Greenberg [54]. On the left are the distance values that are assigned at the first common node if the populations speak different languages. In italics are the populations that speak the language if the name of the language does not match the name of the population.
(PDF)

S26 Fig F_ST_ estimates based on pseudo-non-African allele frequencies. The allele frequencies were estimated by removing African allele frequencies (based on Nuer) to estimate which non-African population is closest to the donor population.
(PDF)

S27 Fig ADZE results with short haplotypes. Five consecutive SNPs have been combined to create short haplotypes. A rank correlation test of the highest sample size (n = 10, Spearman) shows a high correlation (ρ = 0.9050568, p-value < 2.2e-16) with the ADZE result based on the SNPs. Removing the Copts from this increases the correlation slightly (ρ = 0.9240196).
(PDF)

S1 Table Overview of the datasets generated for further analysis. (PDF)

S2 Table Lowest values of f_3_-statistics for targets tested against all possible source combinations. The table is sorted for the statistic value.
(PDF)

S3 Table ALDER results. The table shows the dates of admixture of two source populations to form a target populations.
(PDF)

S4 Table Rolloff results. (PDF)

S5 Table Population sizes of the chimeric datasets. (PDF)

S6 Table Outgroup f_3_ comparing the shared drift of Messiria and source 2. The table is sorted after the f_3_ column.
(PDF)

S7 Table Estimates of admixture times using patterns of LD-decay [34]. The five highest amplitudes of donor population combinations are shown in descending order.
(PDF)
