# Supplementary material for: Northeast African genomic variation shaped by the continuity of indigenous groups and Eurasian migrations
Source: PLoS Genet. 2017 Aug 24;13(8):e1006976. doi: 10.1371/journal.pgen.1006976 (PMC5587336; doi:10.1371/journal.pgen.1006976)
Supplement: S1 Text — (PDF) [file pgen.1006976.s001.pdf]

Supplements

Laboratory procedures

A total of 244 unrelated individuals from 18 populations, previously screened in a microsatellite study (1), were selected to be genotyped on an Illumina Human Omni5MExome SNP-chip.

DNA was extracted from dried blood on Whatman ® FTA ®cards. Sample discs of 1.2mm size were removed from Whatman FTA cards following Whatman FTA Protocol BD09. Between every sample punch, the cutting mat and lab bench were cleaned with 70% Ethanol and DNAaway to avoid cross-contamination. Samples were prepared for DNA analysis following Whatman FTA protocol BD01, using 100µl of FTA Purification Reagent in step 3 and the repeats, instead of 200µl. Samples were amplified using the Illustra Genomiphi V2 DNA Amplification Kit following the protocol from Pinard et al. 2006 (2), after incubating the sample at room temperature with 2.5 µl H_2O for 10 minutes. The amplified DNA was stored at 4 degree Celsius.

DNA concentration was measured using QuBit fluorometric quantitation (54.3 – 370 ng/µL). Amplified products of 25-40µl were sent to the SNP&Seq genotyping platform (NGI and SciLife, Uppsala) for genotyping. A mislabeling led to an exclusion of two samples.

Preparing SNP-chip data

The raw genotyped data contained 4,511,703 SNPs and 243 individuals. The data was processed using PLINK v1.07 (3, 4). Unmapped SNPs, indels and duplicates were removed (13,278 SNPs). The data was filtered for 15% SNP missingness, which excluded 482,923 SNPs and 15% individual's missingness, excluding 11 individuals. The dataset was subsequently filtered for SNPs that were not in Hardy-Weinberg equilibrium (HWE) by grouping populations into three smaller datasets of 6 populations each and excluding SNPs that failed HWE in all three by a significance value of 0.001, which removed 1,174 SNPs from the dataset.

The mislabeled individuals were subsequently excluded and non-autosomal DNA was removed, which left a final dataset of 3,902,437 SNPs and 231 individuals.

Haplotypes and missing data were estimated for the 231 samples using fastPHASE version 1.4.0 (5). The number of haplotype clusters was set to 25 with 25 runs of the EM algorithm. Assumed missingness was set to 10 %. The number of haplotypes sampled from the "posterior" distribution obtained from a particular random start of the EM algorithm was set to 100. After the missing data was imputed, 10 closely related individuals were excluded due to showing IBS values with another individual higher than 0.015 than the median IBS distance of the sample pairs. This excluded 1 Hausa, 6 Bataheen, 1 Shaigia, 1 Gaalien and 1 Zagawa individuals. The high values were always achieved between individuals belonging to the same population. The final dataset contained 221 Individuals from 18 populations (Table 1, Figure 1A).

Reference datasets were cleaned using the same pipeline with following deviations:

- The 1000 Genome dataset (6) was filtered for SNPs that were not in HWE by removing SNPs that failed HWE ($p<0.001$) in all three populations: CEU, YRI, and CHB, tested separately. Imputation was done for separate chromosome arms for chromosomes 1, 2 and 4, due to computational restrictions. Related Individuals were removed according to the 1000 Genome pedigree file. Additionally IBS distance was calculated using plink v.107 (3, 4) and individuals that paired with another with a higher identity by state value than 0.9 were excluded from further analysis.
- The African Genome Variation Project dataset was received filtered for SNPs and related individuals (7). Populations of interest (Amhara, Baganda, Banyarwanda, Barundi, Kalenjin, Kikuyu, Oromo and Somali) were extracted and missing SNPs were imputed using fastPhase (5).

- The Nzime had already been imputed for SNPs, as well as quality filtered for SNPs and related individuals (8).
- We used the gencall dataset for the Egyptian samples (9). SNPs were excluded if they failed HWE at a significance value of p<0.0001. Individuals that scored a higher value than 0.9 in the identity by state distance in pairwise IBD estimation were excluded from further analysis.
- The Ethiopian samples (10) were divided into two groups of 6 populations each to test for SNPs that are not in HWE (p<0.001). Populations that were included in the AGVP dataset were excluded from this dataset after imputation (Amhara, Oromo, and Somali). Individuals marked IBD in the sample file were excluded from further analysis.
- Samples from the Human Genome Diversity Panel (11) were divided into six groups according to their place of origin (Africa, America, Asia, Europe, Middle East and Oceania) to test for SNPs that fail HWE (p<0.001) in all groups. Populations of interest were extracted after imputation of missing data. No individuals had to be excluded due to relatedness.

Merging Datasets

After cleaning and imputing each dataset, we created three merged datasets, which increase in sample size, but decrease in the number of SNPs. To avoid strand issues, A/T and C/G polymorphisms were removed from the datasets. A large dataset containing the Sudanese samples and the Nzime (8) as a west-African comparative population was created. Both datasets were typed on an Illumina HumanOmni5 SNP-chip. After cleaning and merging this dataset retained ~3.5 million SNPS, we call this dataset “dat1”. The second dataset contains the 3.5M SNP-dataset, dat1, as well as selected populations from the African Genome Variation project (7) (Amhara, Baganda, Banyarwanda, Barundi, Kalenjin, Kikuyu, Oromo and Somali), the 1000Genome project (6), and Egyptian data from Pagani et al. 2015 (9). SNP-

names were replaced with chromosomal and bp-position in order to achieve the greatest overlap when merging with the 1000Genome dataset. The merged dataset was down-sampled to include a maximum of 16 individuals per population, to avoid sample size effects. This dataset retained ~1.4 million SNPs and was called “dat2”. The 1.4M SNP-dataset, dat2, was additionally merged with chosen HGDP populations (11) from surrounding regions (Mbuti Pygmies, Biaka Pygmies, Mozabite, Bedouin, Druze and Palestinian) and further Ethiopian populations from Pagani et al. 2012 (10), that have not been included in previously merged datasets (Sudanese, Afar, Esomali, Tygray, Ari Blacksmith, Ari Cultivator, Gumuz, Anuak and Wolayta). Both of these datasets were constructed on hg18, which made it necessary to use liftOver (12) to be able to map them to the hg19 SNPs available in the 1.4M SNP-dataset. This dataset is also down-sampled to a maximum of 16 individuals per population and contains ~220 thousand SNPs and is called “dat3”. An overview of the datasets can be seen in Table S1.

Ancient Individuals

Haploid versions of dat2 and dat3 were merged with data from the bam files of the ancient Ethiopian individual which was found in the Mota cave (Mota) (13) and an ancient European individual from the Linearbandkeramik culture (LBK) (14). We used samtools mpileup v 0.1.19 (15) to extract reads from the SNP position. At each SNP site, a random read with minimum mapping and base quality of 30 was chosen to represent the ancient individual in homozygous state. All transition sites in Mota were coded as missing data as the bam file contains deanimation damage at transition sites which are a characteristic feature of ancient DNA (16). The LBK individual was damage repaired prior to sequencing, so all covered sites could be included in the dataset. After merging to the ancient individuals, all missing SNPs were removed. This dataset was then merged to 17 Ju|’hoansi San individuals from Namibia (17) to serve as an outgroup. After merging and removal of missing SNPs this left 140,302 SNPs when merged with dat2 and 20,125 SNPs with dat3 (Table S1). The dataset including the ancient individuals will be referred to as dat2a and dat3a (a for ancient).

Platform bias

In order to check if the merged datasets are affected by bias, a principal component analysis (PCA) was performed using EIGENSOFT version 5.0.1 (18, 19). PCA was performed without outlier removal, but removing SNPs with an r^2 threshold of 0.4. As seen in Figure S19 there is no obvious effect of a platform bias. Principal Component (PC) 1 is driven by African versus non-African diversity, while PC2 resembles isolation by distance of non-Africans from Africa. American populations appear closer to African and European populations in PC2 due to admixture (20).

Summary Statistics

Heterozygosity and creation of haploidized datasets

Heterozygosity was calculated using Plink v1.07 (3, 4). The Sudanese samples show higher variance than any other population of the global dataset, as seen in the colored populations in Figure S17A. Furthermore, the populations display lower heterozygosity than other populations in the neighboring regions, with similar heterozygosity values as Out-of-Africa populations. This is most likely due to an amplification error. In order to test whether the genotyped samples have experienced allelic drop-out, a chimeric dataset was created. Each individual in the global dataset was randomly haploidized using in-house scripts. Populations were then down-sampled to an even-numbered sample size if required, and subsequently two haploid individuals of a common population were combined to form a chimeric diploid individual. This decreases the sample size by more than half but should the observed patterns be true patterns of the demographic history of the population, we should see them in the chimeric dataset. Heterozygosity was analyzed for this chimeric dataset (Figure S17B). This shows a decrease in variance and higher heterozygosity for the Sudanese populations than seen in the diploid version. The patterns observed in Figure S17 are most likely due to allelic drop-out. In order to deal with the effects of allelic drop-out and to verify results, haploid and chimeric datasets were created for all previously described datasets. While the sample size is decreased by 50% in the haploid dataset, the chimeric datasets lose even more samples, since

population with an odd numbered sample size need to be down-sampled to an even numbered sample size. An overview for the sample sizes of the chimeric datasets can be seen in Table S5. Dataset names will be appended with an 'h' for haploid and 'c' for chimeric.

The Northern Sudanese populations show greater heterozygosity than the Southern populations. Especially the Nilotic populations: Dinka, Nuer, Baria, and Shilluk of South Sudan show low heterozygosity within the Sudanese and East African populations, this can also be observed in the Nilotic populations of previously published datasets (Anuak, Gumuz of Ethiopia and Sudanese). Lowered levels of heterozygosity can also be seen in the Hausa, and the Copts. Low levels of heterozygosity indicate low effective population size, but is more likely due to ascertainment bias, as discussed in Pagani et al. 2015 (9). Populations with less European admixture show lower heterozygosity due to ascertainment of the SNP-panel. This could be the reason of the lower heterozygosity in the Nilotic, Darfurian/Kordofanian populations and the Hausa. The comparatively lower heterozygosity in the Copts compared to the Egyptian population indicates a lower effective population size in the Copts. When comparing the heterozygosity of dat3 to the dataset that retained most SNPs (dat1), the trend of lower heterozygosity in the Nilotic groups is still apparent but less severe (Figure S18). Whereas dat3 shows a greater distribution of values (0.2897027 - 0.3155016), there is less variation in dat1 (0.1819487 - 0.1968244). The Nzime and the Hausa show increased heterozygosity in dat1 compared to dat3, whereas the Copts show a reverse pattern. The ascertainment bias is less severe with more SNPs, this can be explained by the fact that later (denser) Illumina SNP chip versions contained increasing numbers of globally representative SNPs, while earlier, less dense versions were still heavily biased towards populations in the ascertainment panels.

Runs of Homozygosity

Runs of Homozygosity (RoH) represent relatedness in the dataset, the longer the RoH, the more recent the shared parental ancestry and its age (21). By comparing RoH between populations, patterns of the demographic history become apparent.

RoHs were created using plink v.1.07 (3, 4). The following parameters were used: Sliding windows of 50 SNPs with a proportion of 0.05 of overlapping windows that must be called homozygous to define a SNP as in a homozygous segment, allowing one heterozygote site per window. A minimum RoH length of 500kb and 50 SNPs per window was required. The minimum density was set to 50 kb/SNP and the maximum gap between two consecutive SNPs was set to 100 kb to ensure that the length of a RoH is not artificially increased due to local low SNP density. These parameters were similar to parameters used previously in RoH studies (17, 21, 22). The calculation was run for each individual separately. Since imputed datasets were used, the dataset contained no missing data, which was previously noted to be a problem for RoH scans (22). Subsequently a population mean was created for various length categories. Figure S20A shows the RoH calculated for only the Sudanese populations divided into bins of different length. In Figure S21A we can see how the Sudanese populations perform compared to global populations. In the 1000 Genome populations the mean total RoH length decreases with distance from Africa as is discussed in Kirin et al. 2010 (21). The Sudanese populations display higher mean total RoH length than other populations starting in bin 2Mb to 4Mb (Figure S21A). It is apparent that this is an extraordinary amount of very long RoH. This is most likely due to hemizygous deletions (21), rather than a true pattern of the demographic history. As seen in the heterozygosity calculations, the observed pattern is likely due to allelic drop-out.

The test was performed again with dat1c and dat2c, using the same parameters (Figures S20B and 21B). When comparing the number of RoH with the total length of RoH in dat1, it does not follow the linear regression line (Figure S22A). The chimeric dataset shows a decrease in number of RoH and therefore also in total length of RoH, but the results follow a linear regression (Figure S22B). It becomes apparent that the underlying effect of the pattern is most likely allelic drop-out. As the dataset was filtered for related individuals before turning the individuals into chimeric individuals, we do not expect to see RoH longer than 4Mb (21). Dat1c shows fewer long runs than dat2c, which is likely due to the decrease in SNP-density in dat2c.

The Copts display an elevated level of mean total RoH length in the category 0.5 to 1 Mb, which could hint at a smaller effective population size compared to other Sudanese populations. The Egyptian population displays a similar high value of total RoH in this category as the Copts, which is likely due to their shared history.

Fst

Fst was computed using Plink v. 1.9 (23, 24), which calculates Weir and Cockerham's estimate of Fst. Mean Fst values were compared pairwise between populations (Figure 1B, Figure S7A). To validate the results, this was performed on the haploid dataset as well, which correlated significantly (correlation coefficient 0.960-0.999).

To see whether the FST values were driven mostly by European admixture, we calculated pseudo-unadmixed frequencies (25, 26):

$$p^{unadmixed} = \frac{p^{observed} - \propto p^{European}}{1 - \propto}$$

where *p* is the allele frequency, and alpha is the admixture proportion of European ancestry, calculated as an f_4 ratio (see below) by using Mota and Tuscans from Italy (TSI) as sources. To approximate the European frequency we used the allele frequency of TSI. This resulted in less differentiation between the Nilotic to Nubian groups and other Sudanese populations (Figure S7B). It becomes apparent that the differentiation that is seen in Figure S7A is primarily driven by European-like admixture. The Copts show the highest genetic differentiation to the other Sudanese and South Sudanese populations.

We have additionally performed calculations, creating pseudo-non-African allele frequencies by subtracting an African (Nuer) allele frequency for admixed Sudanese populations. The calculations were done on dat2 using the f_4 ratio estimates of dat2 with Nuer versus TSI as African and non-African sources respectively. Admixed populations were chosen if they showed less than 80% African ancestry. The results can be seen in Figure 26. All Sudanese

populations show parallel F_{ST} estimates in a pairwise estimation to non-African populations, which might be hinting at receiving admixture from the same admixture source. The Copts show a smaller differentiation to non-African populations, than the other Sudanese population, which could be due to various reasons. Most likely we overestimated their African ancestry (the Copts show African ancestry of 25.3% compared to 48.3-57.1% of the other admixed Sudanese populations), skewing the pseudo allele frequency calculations. The Copts show a decrease in F_{ST} with the TSI compared to other Sudanese populations, which could be additional admixture. All Sudanese populations show a higher genetic differentiation to Asian populations. The smallest differentiation is seen with the Egyptians, Palestinian and Bedouin groups.

Allelic Richness

Calculations of private alleles and distinct alleles were performed using ADZE version 1.0 (27) on dat1. The Nubians display the highest number of private alleles as well as distinct alleles, while the Nilotes show the lowest values in both (Figure S12A).

Populations were combined in sets of two to investigate shared private alleles (Figure S12B). Within the Arab populations, combinations with Messiria show lower values of shared alleles than other combinations. The highest number of private shared alleles can be seen between Danagla and Halfawieen, which are both Nubian populations. The next highest combinations are seen between Copts and other Sudanese from North-East Sudan, excluding the Hausa. Interestingly combinations with the Copts also include the lowest values (in combination with Nilotes or Darfurian/Kordofanians). The amount of shared alleles resembles a geographic distinction in the populations, possibly driven by the amount of shared alleles due to Eurasian admixture.

To investigate whether we are overestimating the allelic richness in the Sudanese populations that show non-African admixture due to ascertainment bias we performed ADZE on combinations of 5 consecutive SNPs of dat1 and dat2 to create short haplotypes. Short haplotypes are less affected by ascertainment bias (17, 28). In Figure S27, we see that there

is no obvious difference in the allelic richness estimates, but the private allelic richness is more pronounced in the Nubians than before (Spearman rank correlation test rho=0.91). There seems to be no large effect of ascertainment bias on the largeer dataset dat1. However, dat2 is more affected by ascertainment bias (rho=0.22), concurrent with the heterozygosity observation between dat1 and dat2.

Principal component analysis (PCA)

PCA was performed using the smartpca program in EIGENSOFT version 5.01 (18, 19). Analysis was performed including only SNPs that were not in linkage disequilibrium with a threshold of r^2=0.4, and initially without outlier removal.

Dat1

In dat1, which are exclusively African populations, PC1 resembles the variation of West Africa to North-East Africa. This also shows a division between southern and western Sudanese versus northern and eastern Sudanese populations. Higher PC's seems to be driven by variation within populations and outliers in the Nilotic (one Baria individual) and Nubian (two Danagla and two Halfawieen individuals) populations (Figure S13A). After exclusion of these five individuals (Figure S13B) PC1 resembles the variation of West Africa to North-East Africa in this analysis as well. PC2 separates the Sudanese populations from West-African populations such as the recent migrant populations: the Hausa and the Copts. Higher PCs show variation within populations, specifically the Nubians.

In order to validate the results we performed the Principal Component Analysis on the haploid dataset without outlier removal (Figure S14). PC1 and PC2 behave similar to the diploid PCA with outlier removal. PC2 does not seem to be affected by the outliers in the haploid dataset. PC3 shows variation within populations, driven by outliers in the same populations as in the diploid dataset, while PC4 separates outlying Halfawieen individuals.

Dat2

In the 1.4M SNP-dataset PC1 is governed by the variation between African and non-African populations. PC2 resembles the variation of Out-of-Africa populations. PC3 resembles the variation between East-African and West-African populations with the eastern Bantu-speaking populations as an admixed population between those two. PC4 represents the variation found in Native American populations (Figure S15A). African American populations appear as admixed between West-African and European populations, as can be seen in PC1 vs PC2, PC1 vs PC3 and PC2 vs PC3.

The results were validated by performing PCA on the haploidized dataset (Figure S15B) in which the PCs resemble the PCs of the diploid dataset.

Dat3

In the diploid dat3 PC1 resembles variation within Africa to Out-of-Africa (Figure S16A). PC2 is influenced by the variation between Out-of-African populations. PC3 is affected by the variation of African populations, separating Pygmies from West-Africans, Bantu and East African populations. PC4 shows the variation within Native Americans.

When analyzing the haploid version the PCs resemble the same variation as in the diploid dataset (Figure S16B).

Figure S11 shows PC1 versus PC3 of dat3a. The ancient European individual LBK clusters with the modern European Individuals, closest to TSI. The ancient Ethiopian lies between the Ari and the Nilotic populations, which plots them on the same cluster as the Kalenjin.

ADMIXTURE analysis

To evaluate ancestry components we ran ADMIXTURE (29) at default settings with a random seed generated from the clock time, iterating 50 times for each K. Common modes from the replicates were obtained using CLUMPP (30) using the LargeKGreedy algorithm repeating the number of random input orders 1000 times and then visualized with distruct (31).

Dat1 diploid

ADMIXTURE analysis was performed for K=2 to K=7 for the diploid 3.5M SNP-dataset (Figure S1). At K2 the dataset gets divided into Sub-Saharan populations (dark blue) and Saharan populations (teal) with the highest proportion of this component in the Copts. K3 divides the West-African populations (orange) from the other Sub-Saharan Africans. K4 reveals a separate Nubian cluster (blue) while K5 shows a Darfurian/Kordofanian ancestral component (cyan). K6 separates the North-eastern Sudanese (light blue) from the Copts and another Nubian component (dark purple) manifests at K7, which is most prominent in the Mahas.

Dat2 diploid

The 1.4M SNP dataset was analyzed from K=2 through K=15 (Figure S2). At K2 the dataset gets divided into African (yellow) and non-African (green) populations. K3 separates the Asian populations (dark royal blue) from other non-Africans, while K4 distinguishes the Niger-Congo speakers (yellow) from other the Nilo-Saharan and Afro-Asiatic speakers (dark blue). At K5 the Afro-Asiatic and Nubian populations are distinguished (teal). K6 assigns a component to Native American populations (dark green). At K7 the Cushitic populations become distinct (pink). The Indian population becomes distinct (light purple) at K8. At K9 a blue component appears in the Darfurians and Kordofanians and most northern and north-eastern Sudanese. At K10 this blue component is exclusive to the Nubians, while an orange component appears in the West-African populations and separates them from other Niger-Congo speakers. K11 distinguishes the Darfurian/ Kordofanian populations (cyan) and K12 the Japanese (dark royal blue). K13 introduces a light blue component in the Northern Sudanese populations while K14 shows an exclusive component to the Nuba (royal blue). K15 separates the Finns from the other Europeans.

Dat3 diploid

We ran K2 through K20 for dat3 (Figure S3). At K2 the dataset is divided into Africans (dark blue) and non-Africans (green). K3 divides the East Asian populations (purple) from the other populations, K4 distinguished the Niger-Congo speakers and Pygmies (orange) from the Afro-

Asiatic and Nilo-Saharan speakers. K5 assigns a cluster to American populations (dark green) while K6 divides the Afro-Asiatic populations and Nubians (brown) from the other African populations. K7 extracts the Mbuti Pygmies (salmon) and K8 the Ari (bright pink). K9 distinguished the Indian population (light purple) from the European and Asian. At K10 a pink component shows up in most Afro-Asiatic populations that is most prominent in Cushitic speakers. K11 assigns a separate cluster to the Biaka Pygmies (dark salmon), K12 to the Gumuz (red) and K13 to the Mozabite (tan brown). AT K14 and K15 the Druze get assign a specific cluster (olive) as well as the Northern and Eastern Sudanese Populations (blue) that is most prominent in the Nilotic populations. K16 separates the Japanese (dark royal blue) from the East Asians. K17 distinguishes the Darfurians and Kordofanians (cyan) from the Nilotes. K18 assigns a distinct cluster to the Copts (teal) while K19 distinguishes the Bantu-speaking populations (yellow). K20 separates the Ari Cultivator (light pink) from the Ari Blacksmith.

Haploid verifications

To validate the results, ADMIXTURE analysis was performed on the haploid versions of the dataset. The haploid version of dat1 shows similar patterns as the diploid for ADMIXTURE analysis (Figure S4). It was also performed for K=2 through K=7, although at K6 and K7 no common nodes can be found. The same patterns arise however for K2 through K5 as in the diploid version.

ADMIXTURE for dat2h was also performed for K=2 through K=15 (Figure S5). However at K=14 no common nodes are found and K=15 can only find a maximum of 2 common modes. This analysis matches the ADMIXTURE analysis of the diploid 1.4M SNP-dataset up to K=8. At K9 the West-Africans become distinct and the blue component that separates the Nubians comes in at K10. K11 distinguishes the Darfurians and Kordofanians (cyan) from the Nilotes. At K12 the Maasai get their own cluster assigned (light yellow), which disappears again at K13, which instead has separated the Japanese (dark royal blue) from the Asians and the Northern Sudanese (light blue) from the Copts and Egyptians (teal). K14 shows a separate cluster for

the Kikuyu (bright yellow) that disappears again at K15 which shows a separate cluster for Puerto Ricans (light green) and Maasai (light yellow) instead.

The dat3h ADMIXTURE analysis was performed for K2 through K20 (Figure S6). K2 through K5 assigns clusters in the same order as the diploid dataset, while K6 assigns a separate cluster to the Mbuti (salmon) and K7 divides the Afro-Asiatic populations and Nubians (brown) from the other African populations. K8 distinguished the Ari speakers (bright pink) and K9 assigns a cluster to most Afro-Asiatic populations that is most prominent in the Cushitic speakers (pink). At K10 distinguishes the Indians (light purple), K11 the Gumuz (red), K12 the Biaka Pygmies (dark salmon), and K13 the Mozabite (tan). At K14 the Japanese (dark royal blue) and the Darfurians and Kordofanians (cyan) show up as separate clusters but the Gumuz (red) component disappears. At K15 the red component returns along with a distinct component for the Druze (olive), but the Japanese component disappears. This gets resolved at K16, where all previously mentioned components are visible. K17 marks the first appearance of a Bantoid (yellow) and a Nubian (blue) component, but loses the Darfurian/Kordofanian component. The Nubian component is not seen in K18 and K19, which both show the Darfurian/Kordofanian component again. K18 introduces a Coptic component (teal) while K19 distinguishes the Maasai (light yellow). This gets resolved partly in K20, the Nubian component reappears but the Bantu-speaker component is lost instead, which results in a distinction of the Ari Cultivators (light pink) from the Ari Blacksmiths.

Mota and LBK

An ADMIXTURE analysis was performed of Mota and LBK together with a haploidized version of dat3a (Figure S24). The Mota individual shares components with the Ari and the Gumuz. The LBK individual shares components with the Europeans, Bedouins and Druze. The component sharing is likely due to the ancestral state of the samples to these populations.

f_3

Admixture was formally tested using f_3 statistics as implemented in the Admixtools package (32). f_3 tests were performed on dat3. All population combinations were tested as source

populations to the target Sudanese and South Sudanese populations. Only f3 values that were significant (Z-score lower than -2.4) were considered. The analysis was also performed with the corresponding haploid dataset, dat3h. The results decreased in significance but correlated significantly (correlation coefficient 0.99967).

Outgroup-F_3

In order to test which of the populations is closest related to Mota we performed an outgroup f_3 analysis, using the Ju|'hoansi San from Namibia as the outgroup (32). As has been shown previously the Ari show increased shared drift with Mota (13) (Figure S23). The highest value in any of the novel Sudanese and South Sudanese populations is found in the Beni-Amer, that also show the highest value of the East African Somali component of the Sudanese and South Sudanese populations in the ADMIXTURE analyses.

We furthermore tested shared drift in the Arab Messiria population. The Messiria share the longest drift with the Sudanese Arab populations, compared to Nilotic and Darfurian/Kordofanian populations (Table S6).

Dating Admixture

We used LD decay patterns to investigate and estimate the time of admixture. We used Malder (33), and analyzed dat3 to investigate whether the LD-decay is more consistent with more than one admixture event. A genetic map was calculated for the dataset, using the combined genetic map of the 1000 Genome project. All populations were allowed as possible source population. The best fit of admixture sources for all investigated targets appeared to be a Nilotic population mixing with a non-African (mostly European) population (Table S7), for populations that previously showed consistent decay rates with two source populations in the ALDER analyses. In three target populations there were two admixture events detected. We found additional admixture events in various populations where the Alder approach failed to detect consistent decay curves (Baria, Nuer, Hausa, Nuba, Zagawa, Table S3). The Baria and Nuba

show unclear signals for the admixture source, showing a complicated admixture pattern of an African and a Eurasian source. We did not find admixture in the Dinka and the Copts.

Alder and Admixtools (Rolloff analysis, where Alder analysis failed) were used to infer additional admixture dates (32). Default parameters were implemented. Various LD decay curves were estimated using different populations as the two parental reference populations (Table S3 and S4). The standard error was estimated with a jackknife procedure. Generations were converted to years using 30 years per generation.

Correlations with Geography and Linguistic affiliations

We correlated F_{ST} distances with geography and linguistic affiliation. A Mantel test was performed using the ade4 package in R, with 9999 replicates.

To compare the F_{ST} distances to linguistic distances we assigned linguistic distances as seen in Figure S25. If the populations speak the same language a value of zero was assigned. The linguistic classification is according to Greenberg (34). The Gemar speak a form of Standard Arabic, but have historically spoken an Eastern Saharan language that is now extinct. They were classified as Eastern Saharan speakers for this analysis, with a small distance to the Zaghawa. (Personal communication with Mohammed El-Zaki Abu-Baker 2014-02-05, 11:00 GMT). The Nuba were excluded from this analysis, due to their linguistic complexity. Fst correlates with the linguistic affiliation by r= 0.2756918 (p=0.0083).

Geographical distances were calculated with greater circle distance to account for the curvature of the Earth. F_{ST} was compared both to sampling location as well as to a mean point of the habitational area of the populations. F_{ST} correlates with the mean point of the habitational areas by r=0.3908911 (p=0.0012). The Messiria were excluded from the comparison with Sampling Locations (see Babiker 2011). Sampling Location and Fst correlate by r= 0.3919844 (p=0.0013). The Copts and the Hausa are recent migrants to the Sudan. Exclusion of the Copts and the Hausa increases the geographic correlation (r=0.67, p-value: 2e-04) with the mean

habitational point as well as with the sampling location (r=0.6361352, p-value: 3e-04) but not with linguistic affiliation (0.2765088 p-value: 0.0167).

When comparing the F_{ST} values of the pseudo-unadmixed allele frequencies, genetic distance correlates slightly stronger with linguistic affiliation (r=0.3928124, p-value:2e-04) than geographic distance (r=0.3521214, p-value: 0.0012 for habitational mean, r= 0.2966525, p-value: 0.0089 for sampling location).

We furthermore performed partial Mantel tests to control for the other factor. We used the R package vegan with 9999 permutations and Pearson correlation coefficient. We removed the Messiria and the Nuba from the analysis, due to their unknown sampling site and linguistic classification respectively. Genetic distance (F_{ST}) was compared to geographic distance (sampling sites) and linguistic distance. Genetic distance correlates more with geographic distance (r=0.3113) than linguistic distance (r=0.2389) when accounting for the respective third factor. When additionally removing the recent migrant population (Hausa and Copt) the correlation between genetic and geographic distance increases (r= 0.5842), while the correlation between genetic and linguistic distance decreases (r=0.1588).

D-Statistics

D-statistics were calculated using Admixtools (32) to test consistency of the data with various tree topologies. Standard errors were calculated using a block jackknife of 0.5 Mbp. D((Outgroup,X);(Y,Z)) tests the tree topology ((Outgroup,X);(Y,Z)). Using Ju|'hoansi as an outgroup we can interpret significantly positive values as a rejection of the tree due to an excess of shared alleles between X and Z while significantly negative values indicate affinity between X and Y, which is not consistent with the tree topology ((Outgroup,X);(Y,Z)) (Figure S8).

F_4-Ratio

f_4 ratios were calculated using Admixtools to estimate admixture proportions based on f statistics (32). Using the ancient Ethiopian, Mota, as a reference for an unadmixed East

African, Eurasian back-admixture was estimated as 1-f_4(CHB,GBR;X,LBK)/f_4(CHB,GBR;Mota,LBK) similar to the statistics used in (13)(Figure S9A).

Furthermore, we merged the information for three archaic hominins, two Neanderthals (Prüfer et al 2014) and one Denisovan (Meyer et al 2012) with dat2a using the same approach as described for the ancient humans above. Then the f4-ratio f_4(Denisovan,Altai Neanderthal; Juhoansi, X))/f_4(Denisovan, Altai Neanderthal; Juhoansi, Mezmaiskaya Neanderthal) was used to explore the Neanderthal contribution to East African populations (Figure 9B). Neanderthal and Eurasian proportions are highly correlated (0.92349), suggesting that the Eurasian back-admixture is the source of all Neanderthal ancestry in North-East Africa (Figure 9C) as shown previously by Haber et al. 2016 (35).

References

1. Babiker HM, Schlebusch CM, Hassan HY, Jakobsson M. Genetic variation and population structure of Sudanese populations as indicated by 15 Identifiler sequence-tagged repeat (STR) loci. Investigative genetics. 2011;2(1):12.
2. Pinard R, de Winter A, Sarkis GJ, Gerstein MB, Tartaro KR, Plant RN, et al. Assessment of whole genome amplification-induced bias through high-throughput, massively parallel whole genome sequencing. Bmc Genomics. 2006;7(1):216.
3. Purcell S, Neale B, Todd-Brown K, Thomas L, Ferreira MA, Bender D, et al. PLINK: a tool set for whole-genome association and population-based linkage analyses. The American Journal of Human Genetics. 2007;81(3):559-75.
4. Purcell S. Plink v1.07 http://pngu.mgh.harvard.edu/purcell/plink/2010.
5. Scheet P, Stephens M. A fast and flexible statistical model for large-scale population genotype data: applications to inferring missing genotypes and haplotypic phase. American journal of human genetics. 2006;78(4):629-44.
6. 1000 Genomes Project Consortium, Auton A, Brooks LD, Durbin RM, Garrison EP, Kang HM, et al. A global reference for human genetic variation. Nature. 2015;526(7571):68-74.
7. Gurdasani D, Carstensen T, Tekola-Ayele F, Pagani L, Tachmazidou I, Hatzikotoulas K, et al. The African Genome Variation Project shapes medical genetics in Africa. Nature. 2015;517(7534):327-32.
8. Sjöstrand AE. Origins and Adaptation in Humans: A Case Study of Taste and Lifestyle. Acta Universitatis Upsaliensis Uppsala University; 2015.
9. Pagani L, Schiffels S, Gurdasani D, Danecek P, Scally A, Chen Y, et al. Tracing the route of modern humans out of Africa by using 225 human genome sequences from Ethiopians and Egyptians. American journal of human genetics. 2015;96(6):986-91.
10. Pagani L, Kivisild T, Tarekegn A, Ekong R, Plaster C, Gallego Romero I, et al. Ethiopian genetic diversity reveals linguistic stratification and complex influences on the Ethiopian gene pool. American journal of human genetics. 2012;91(1):83-96.

11. Cann HM, de Toma C, Cazes L, Legrand MF, Morel V, Piouffre L, et al. A human genome diversity cell line panel. Science. 2002;296(5566):261-2.
12. Kuhn RM, Haussler D, Kent WJ. The UCSC genome browser and associated tools. Briefings in Bioinformatics. 2012.
13. Gallego Llorente M, Jones ER, Eriksson A, Siska V, Arthur KW, Arthur JW, et al. Ancient Ethiopian genome reveals extensive Eurasian admixture throughout the African continent. Science. 2015;350(6262):820-2.
14. Lazaridis I, Patterson N, Mittnik A, Renaud G, Mallick S, Kirsanow K, et al. Ancient human genomes suggest three ancestral populations for present-day Europeans. Nature. 2014;513(7518):409-13.
15. Li H, Handsaker B, Wysoker A, Fennell T, Ruan J, Homer N, et al. The sequence alignment/map format and SAMtools. Bioinformatics. 2009;25(16):2078-9.
16. Sawyer S, Krause J, Guschanski K, Savolainen V, Pääbo S. Temporal patterns of nucleotide misincorporations and DNA fragmentation in ancient DNA. PloS one. 2012;7(3):e34131.
17. Schlebusch CM, Skoglund P, Sjodin P, Gattepaille LM, Hernandez D, Jay F, et al. Genomic variation in seven Khoe-San groups reveals adaptation and complex African history. Science. 2012;338(6105):374-9.
18. Patterson N, Price AL, Reich D. Population structure and eigenanalysis. PLoS genetics. 2006;2(12):e190.
19. Price AL, Patterson NJ, Plenge RM, Weinblatt ME, Shadick NA, Reich D. Principal components analysis corrects for stratification in genome-wide association studies. Nature genetics. 2006;38(8):904-9.
20. Bryc K, Auton A, Nelson MR, Oksenberg JR, Hauser SL, Williams S, et al. Genome-wide patterns of population structure and admixture in West Africans and African Americans. Proceedings of the National Academy of Sciences. 2010;107(2):786-91.
21. Kirin M, McQuillan R, Franklin CS, Campbell H, McKeigue PM, Wilson JF. Genomic runs of homozygosity record population history and consanguinity. PLoS One. 2010;5(11):e13996.
22. Henn BM, Gignoux CR, Jobin M, Granka JM, Macpherson J, Kidd JM, et al. Hunter-gatherer genomic diversity suggests a southern African origin for modern humans. Proceedings of the National Academy of Sciences. 2011;108(13):5154-62.
23. Chang CC, Chow CC, Tellier L, Vattikuti S, Purcell SM, Lee JJ. Second-generation PLINK: rising to the challenge of larger and richer datasets. Gigascience. 2015;4(7).
24. Purcell S, Chang CC. Plink v1.9 https://www.cog-genomics.org/plink22015 [Available from: http://pngu.mgh.harvard.edu/purcell/plink/.
25. Bhatia G, Patterson N, Pasaniuc B, Zaitlen N, Genovese G, Pollack S, et al. Genome-wide comparison of African-ancestry populations from CARe and other cohorts reveals signals of natural selection. The American Journal of Human Genetics. 2011;89(3):368-81.
26. Huerta-Sánchez E, DeGiorgio M, Pagani L, Tarekegn A, Ekong R, Antao T, et al. Genetic signatures reveal high-altitude adaptation in a set of Ethiopian populations. Molecular biology and evolution. 2013;30(8):1877-88.
27. Szpiech ZA, Jakobsson M, Rosenberg NA. ADZE: a rarefaction approach for counting alleles private to combinations of populations. Bioinformatics. 2008;24(21):2498-504.
28. Conrad DF, Jakobsson M, Coop G, Wen X, Wall JD, Rosenberg NA, et al. A worldwide survey of haplotype variation and linkage disequilibrium in the human genome. Nat Genet. 2006;38(11):1251-60.

29. Alexander DH, Novembre J, Lange K. Fast model-based estimation of ancestry in unrelated individuals. Genome research. 2009;19(9):1655-64.
30. Jakobsson M, Rosenberg NA. CLUMPP: a cluster matching and permutation program for dealing with label switching and multimodality in analysis of population structure. Bioinformatics. 2007;23(14):1801-6.
31. Rosenberg NA. DISTRUCT: a program for the graphical display of population structure. Molecular Ecology Notes. 2004;4(1):137-8.
32. Patterson N, Moorjani P, Luo Y, Mallick S, Rohland N, Zhan Y, et al. Ancient admixture in human history. Genetics. 2012;192(3):1065-93.
33. Pickrell JK, Patterson N, Loh PR, Lipson M, Berger B, Stoneking M, et al. Ancient west Eurasian ancestry in southern and eastern Africa. Proc Natl Acad Sci U S A. 2014;111(7):2632-7.
34. Greenberg JH. The languages of Africa: Indiana Univ.; 1963.
35. Haber M, Mezzavilla M, Bergström A, Prado-Martinez J, Hallast P, Saif-Ali R, et al. Chad Genetic Diversity Reveals an African History Marked by Multiple Holocene Eurasian Migrations. The American Journal of Human Genetics. 2016.
