# Supplementary figures and images for: Northeast African genomic variation shaped by the continuity of indigenous groups and Eurasian migrations
Source: PLoS Genet. 2017 Aug 24;13(8):e1006976. doi: 10.1371/journal.pgen.1006976 (PMC5587336; doi:10.1371/journal.pgen.1006976)

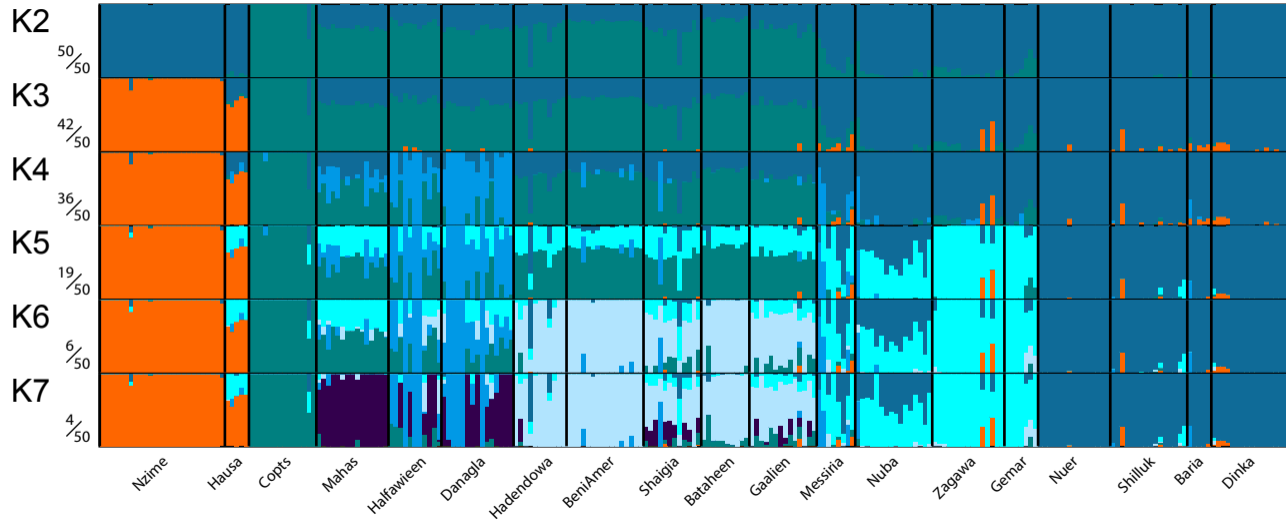

Supplement: S1 Fig — The cluster number can be found on the left along with the amount of iterations that support this cluster out of 50 (CLUMPP) [52, 53]. (PDF) [file pgen.1006976.s002.pdf]

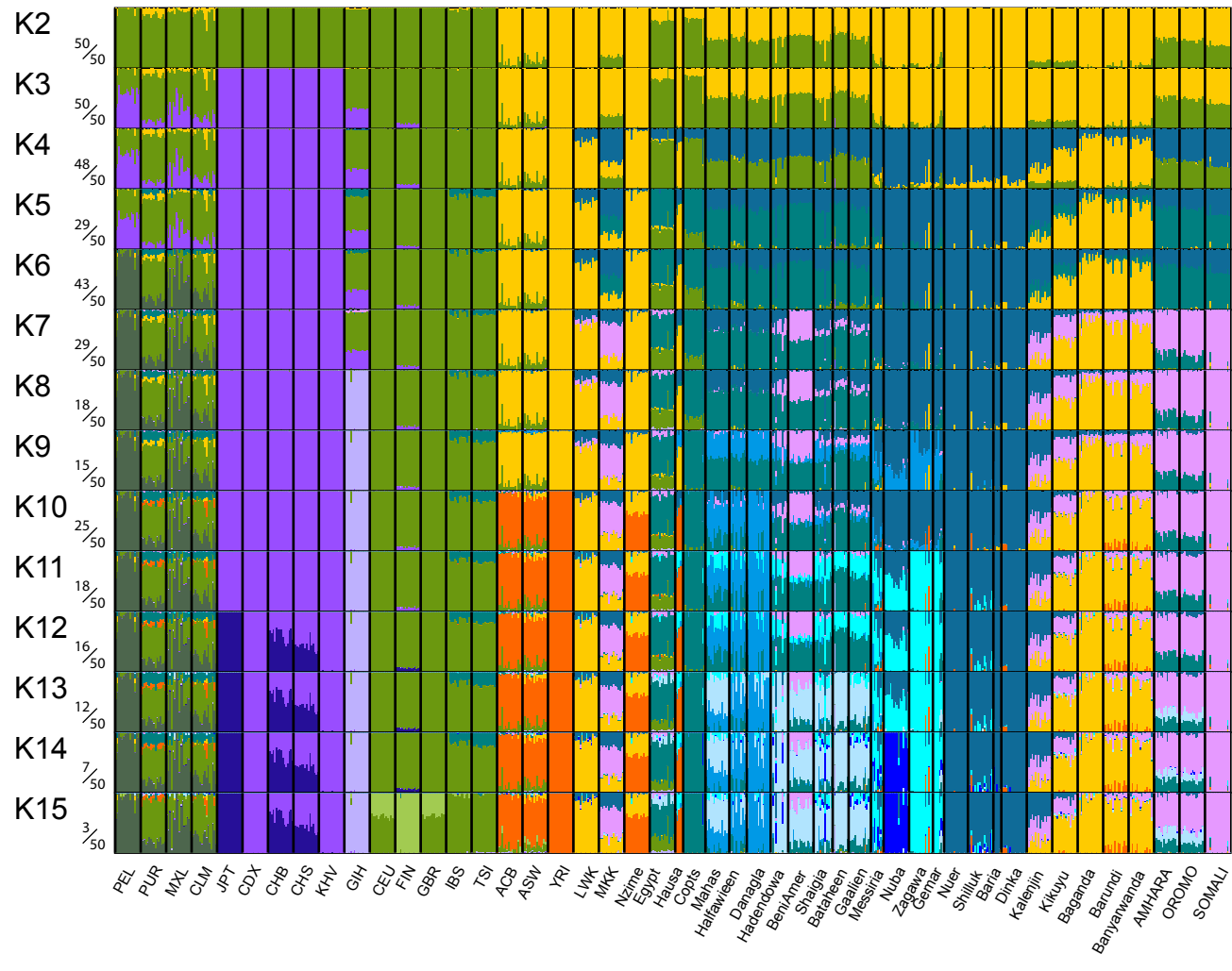

Supplement: S2 Fig — The cluster number can be found on the left along with the amount of iterations that support this cluster out of 50 (CLUMPP) [52, 53]. (PDF) [file pgen.1006976.s003.pdf]

K2

50

K3

50

K4

50

# K5

43

## K6

29

K/

30

K8

160

K9  
20

K14

30

K15

15

K11

14

K13

K14

•

K15

16

K1e

23

K15

10

K18

11

K19

10

K20

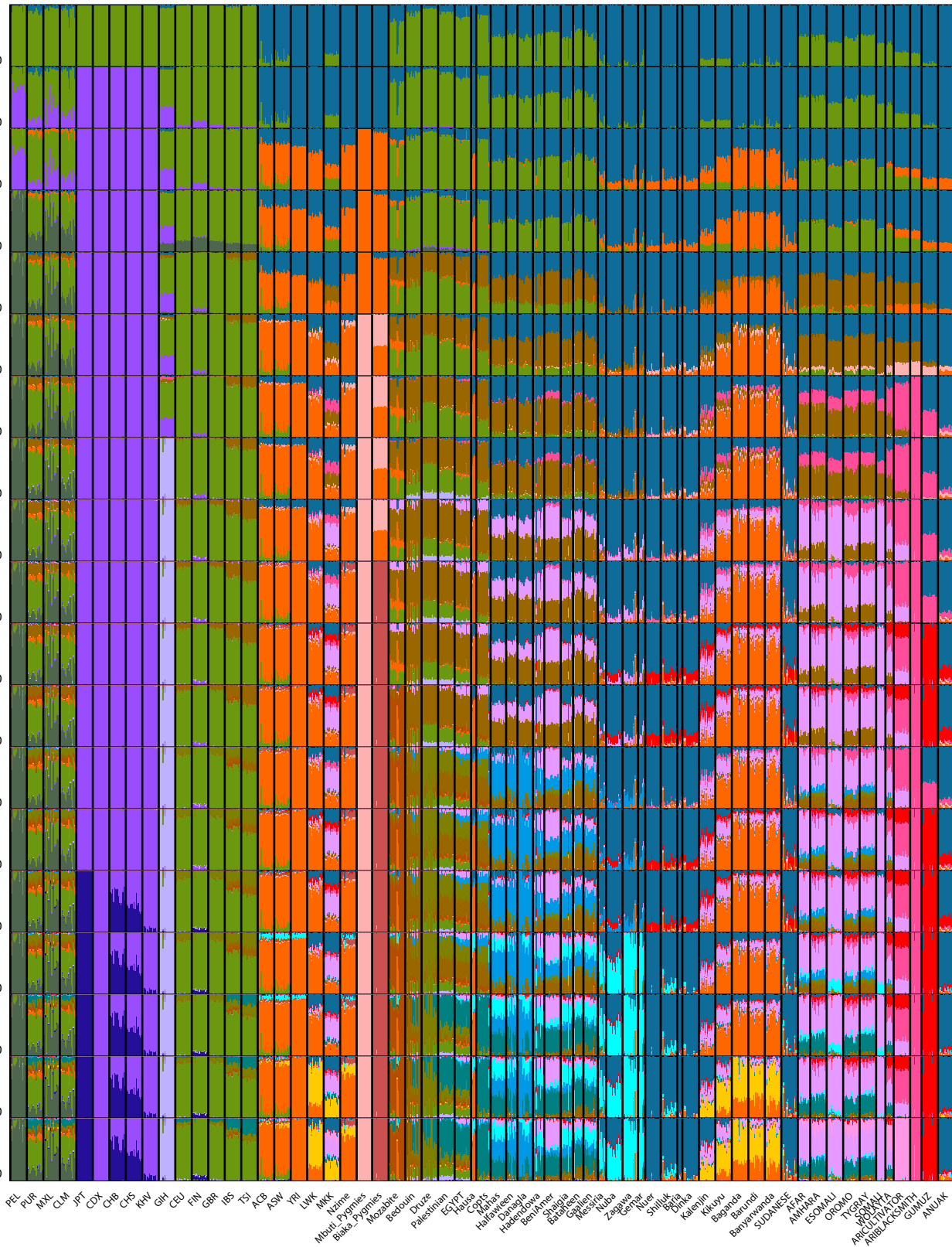

Supplement: S3 Fig — The cluster number can be found on the left along with the amount of iterations that support this cluster out of 50 (CLUMPP) [52, 53]. (PDF) [file pgen.1006976.s004.pdf]

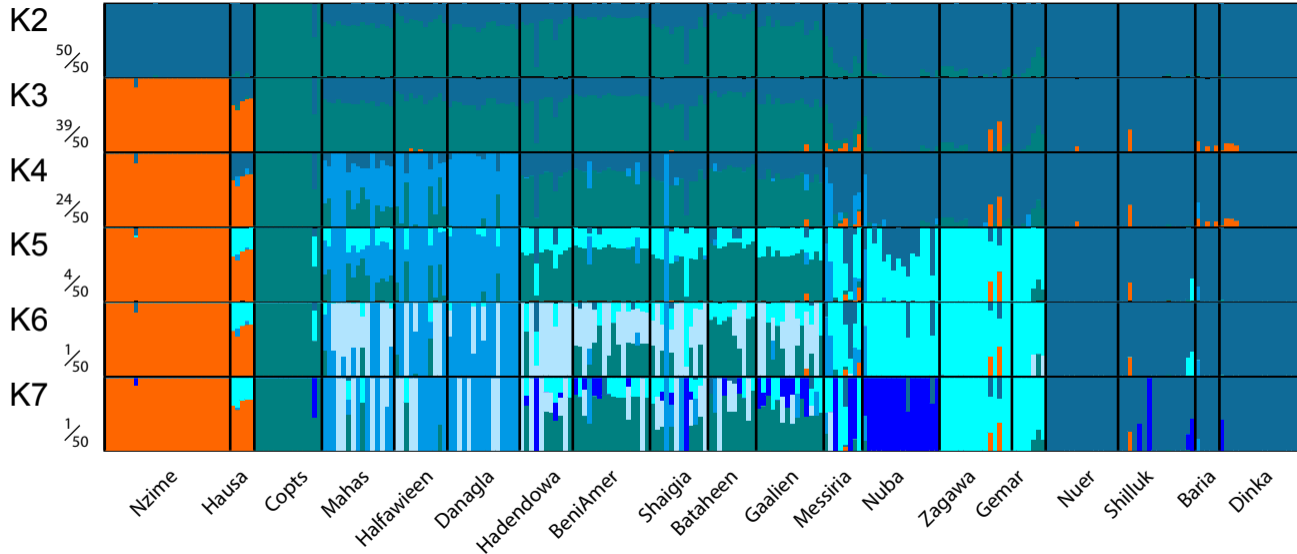

Supplement: S4 Fig — The cluster number can be found on the left along with the amount of iterations that support this cluster out of 50 (CLUMPP) [52, 53]. (PDF) [file pgen.1006976.s005.pdf]

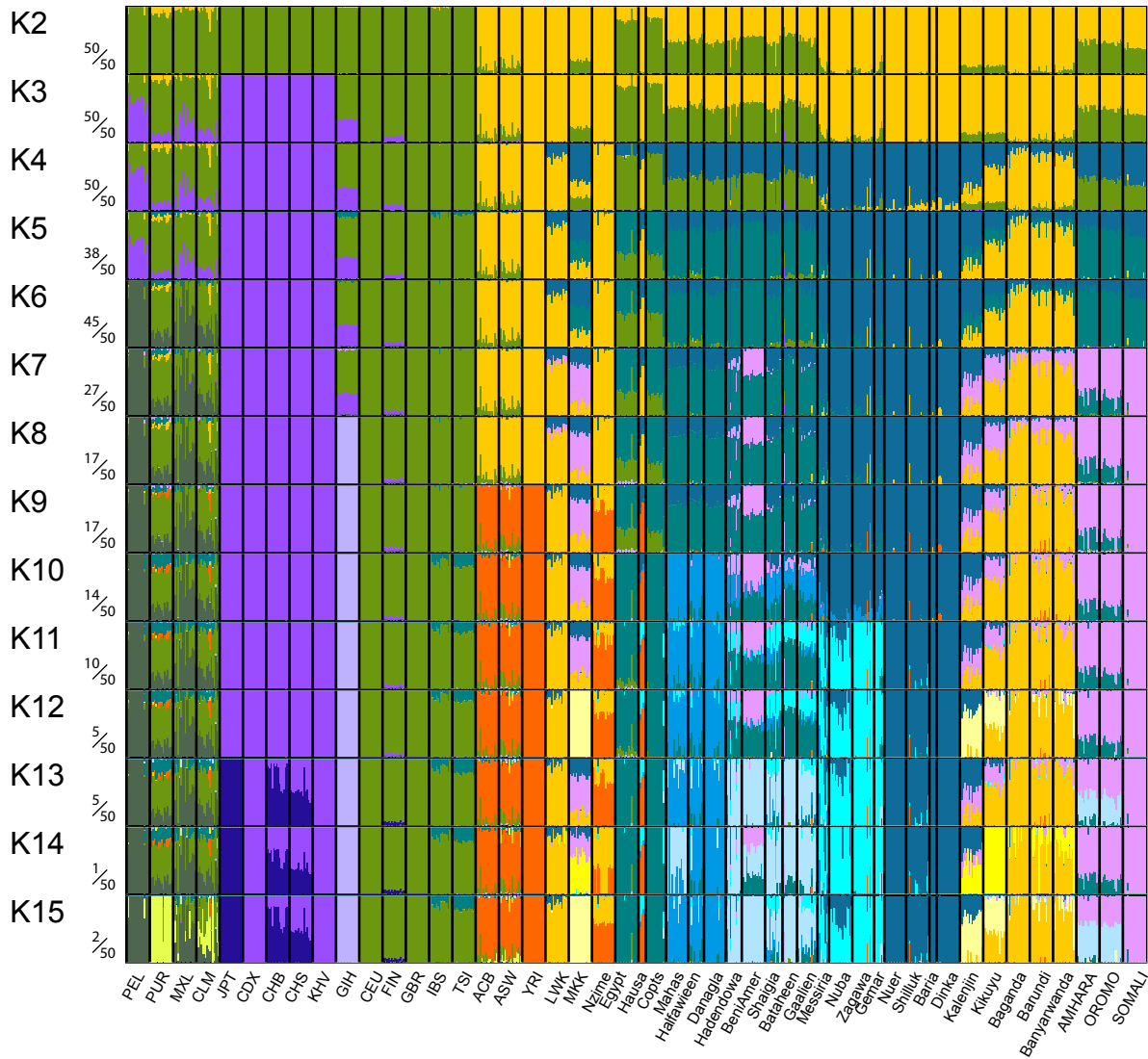

Supplement: S5 Fig — The cluster number can be found on the left along with the amount of iterations that support this cluster out of 50 (CLUMPP) [52, 53]. (PDF) [file pgen.1006976.s006.pdf]

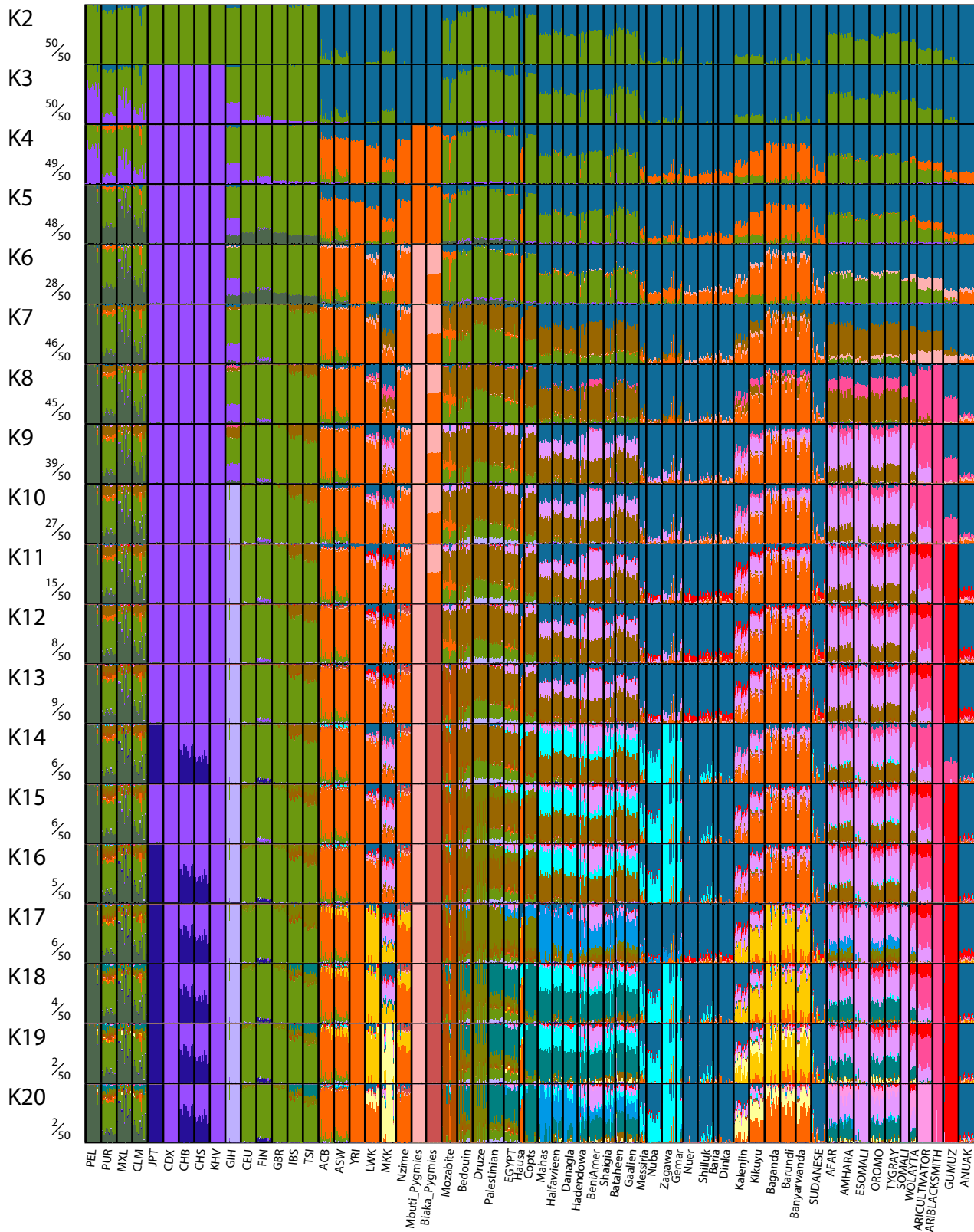

Supplement: S6 Fig — The cluster number can be found on the left along with the amount of iterations that support this cluster out of 50 (CLUMPP) [52, 53]. (PDF) [file pgen.1006976.s007.pdf]

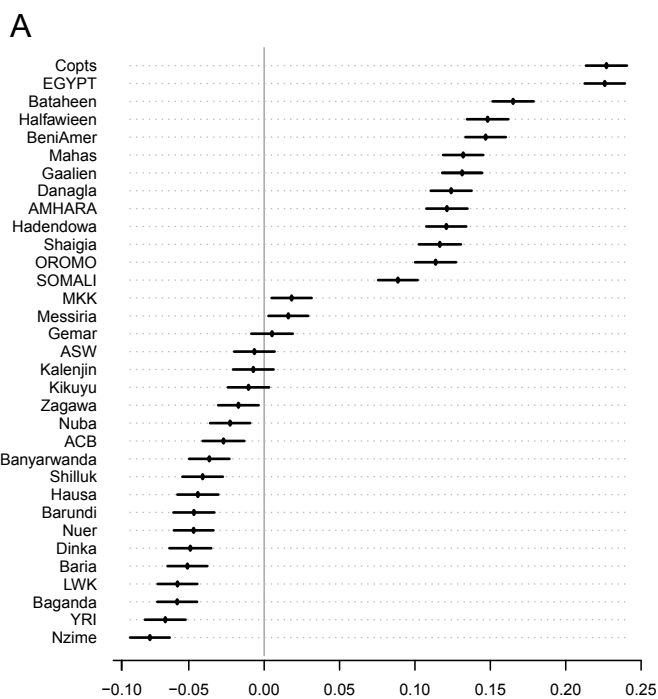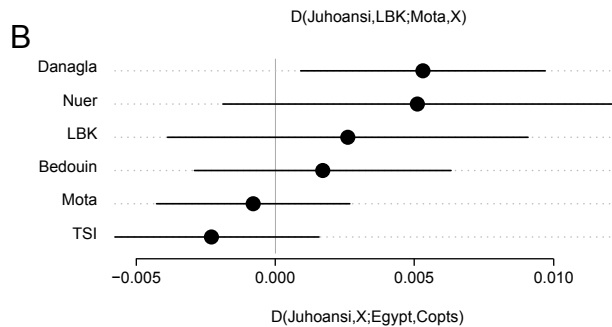

Supplement: S8 Fig — (A) Results for D(Ju|’hoansi, LBK; Mota, X) to account for non-African admixture in population X, where X is the population on the y-axis. (B) Results for D(Ju|’hoansi, X; Egypt, Copt) to investigate whether Egyptians or Copts received more admixture of source X, where X is the population on the y-axis. (PDF) [file pgen.1006976.s009.pdf]

A

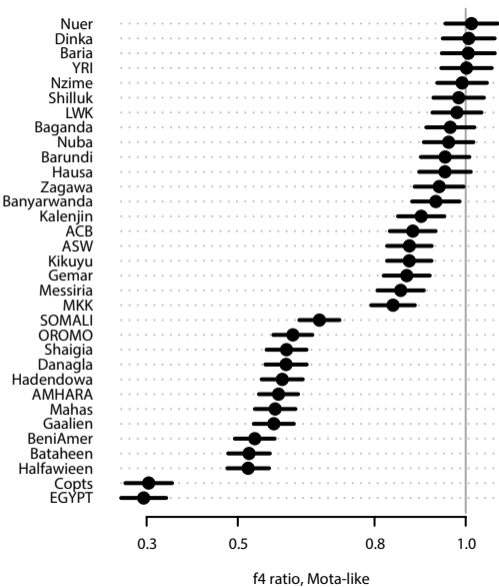

B

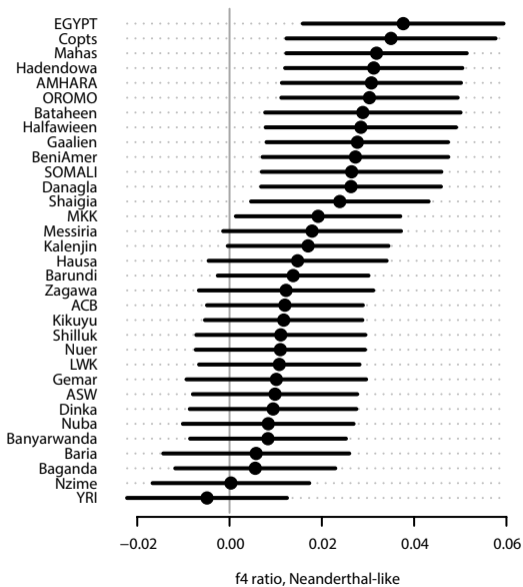

C

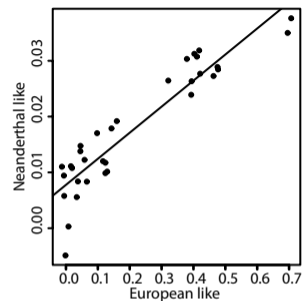

Supplement: S9 Fig — (A) shows the Mota-like proportion in the populations on the Y axis. Horizontal bars display 2SE. (B) shows the Neanderthal-like proportion in the populations on the Y axis. Horizontal bars display 2SE. (C) Correlation of the Neanderthal and European proportions (r = 0.925). The European proportion was calculated as 1- Mota-like proportion. (PDF) [file pgen.1006976.s010.pdf]

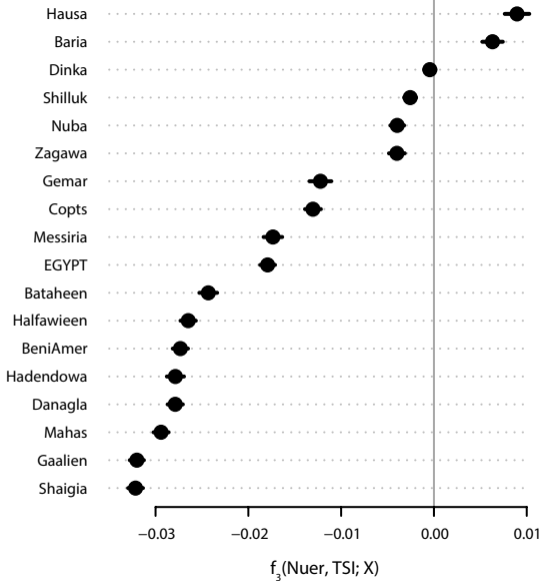

Supplement: S10 Fig — On the Y axis are the target populations. The lines around the circle show 2SE. (PDF) [file pgen.1006976.s011.pdf]

A

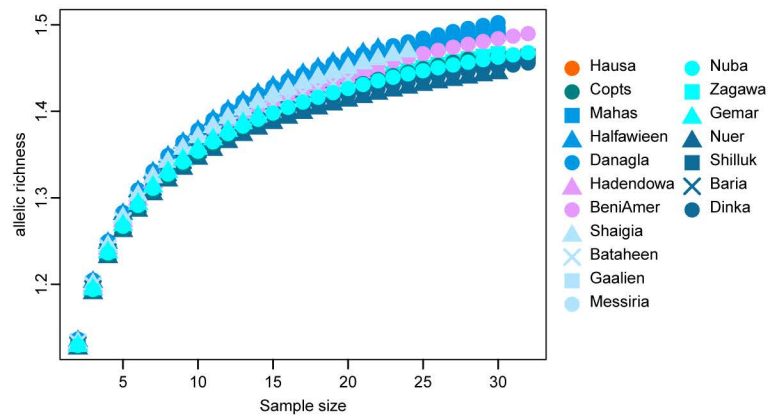

B

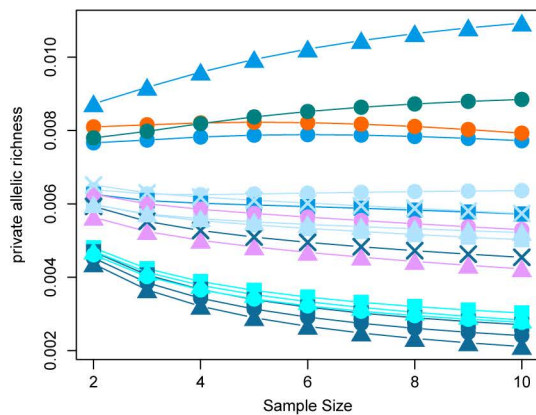

C

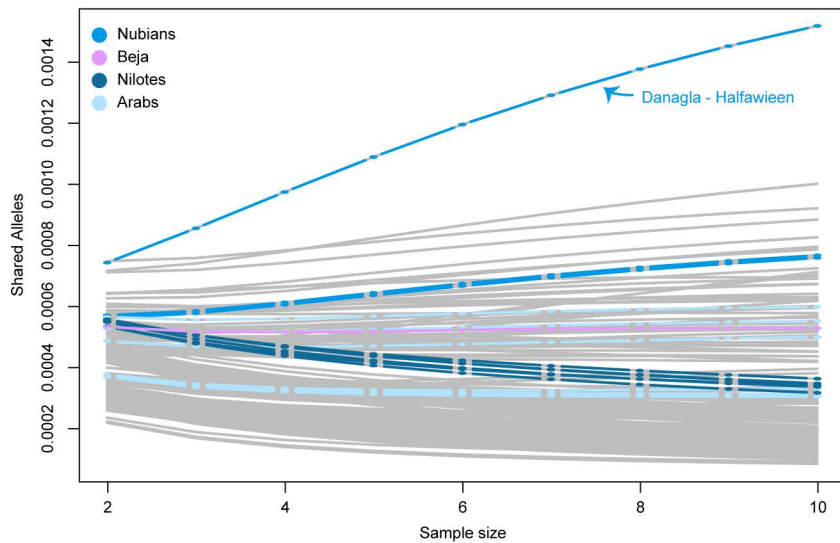

Supplement: S12 Fig — (A) Allelic richness and (B) private allelic richness for the Sudanese and the South Sudanese populations computed on a non-merged dataset using ADZE [5]. Shared Alleles (C) between populations within an ethnic group are highlighted. All possible pairwise combinations are shown in gray. The highest amount of shared alleles is found between the Danagla and Halfawieen. (PDF) [file pgen.1006976.s013.pdf]

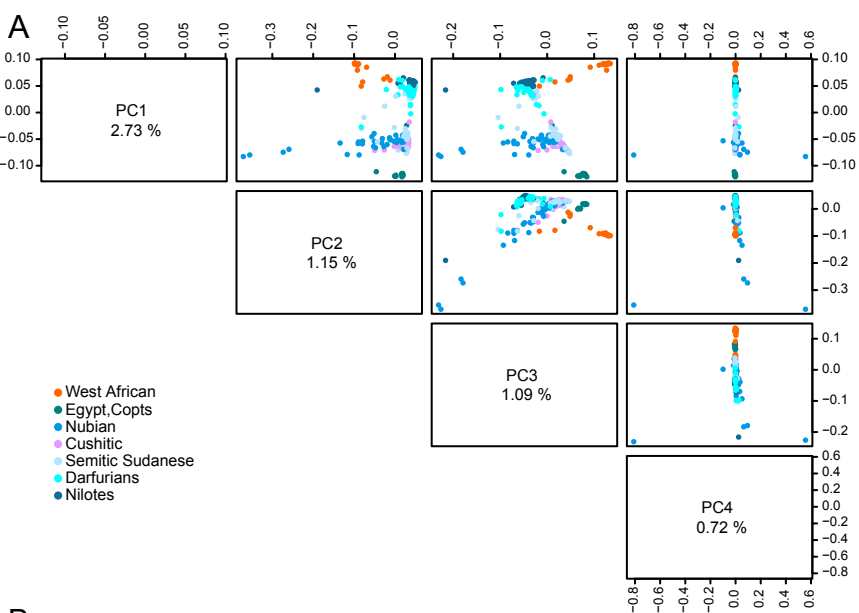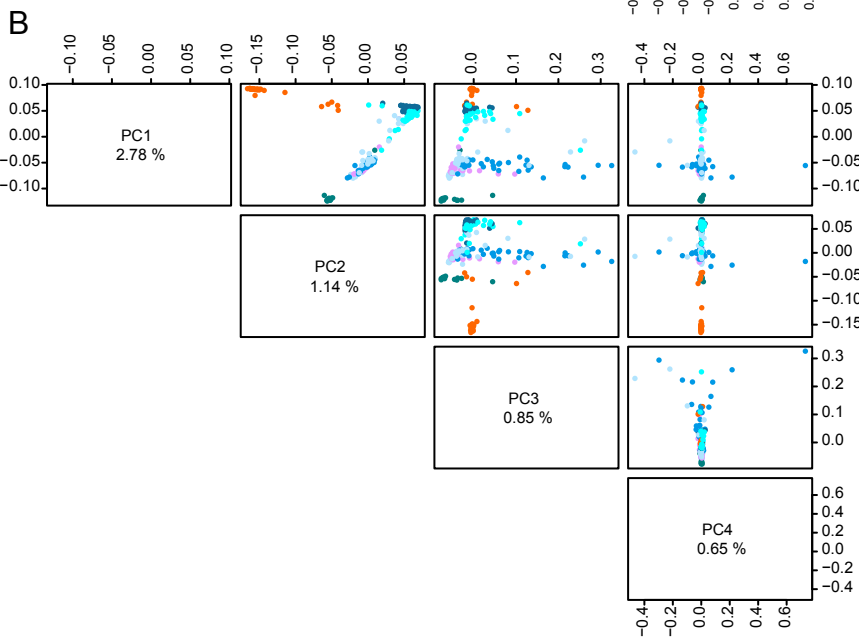

Supplement: S13 Fig — (A) No outlier removal. (B) Five outliers removed. (PDF) [file pgen.1006976.s014.pdf]

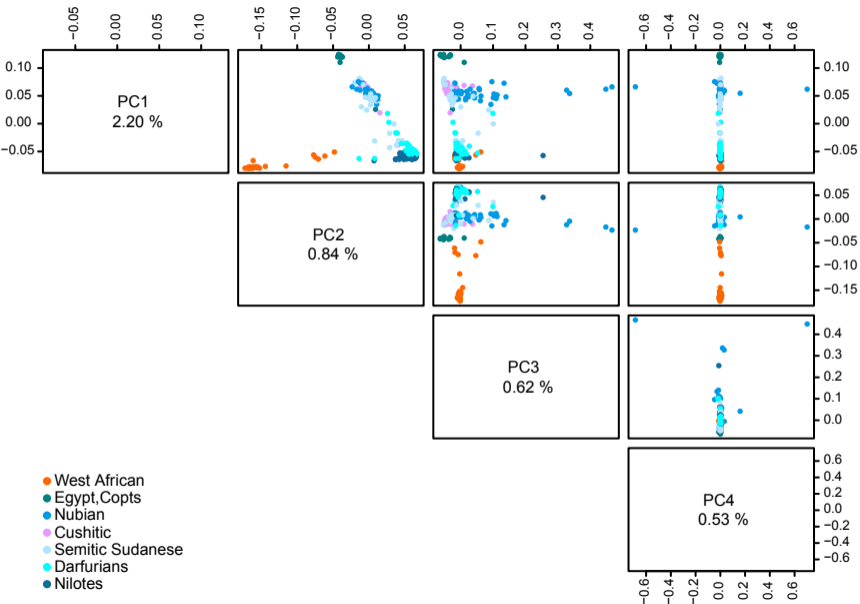

Supplement: S14 Fig — (PDF) [file pgen.1006976.s015.pdf]

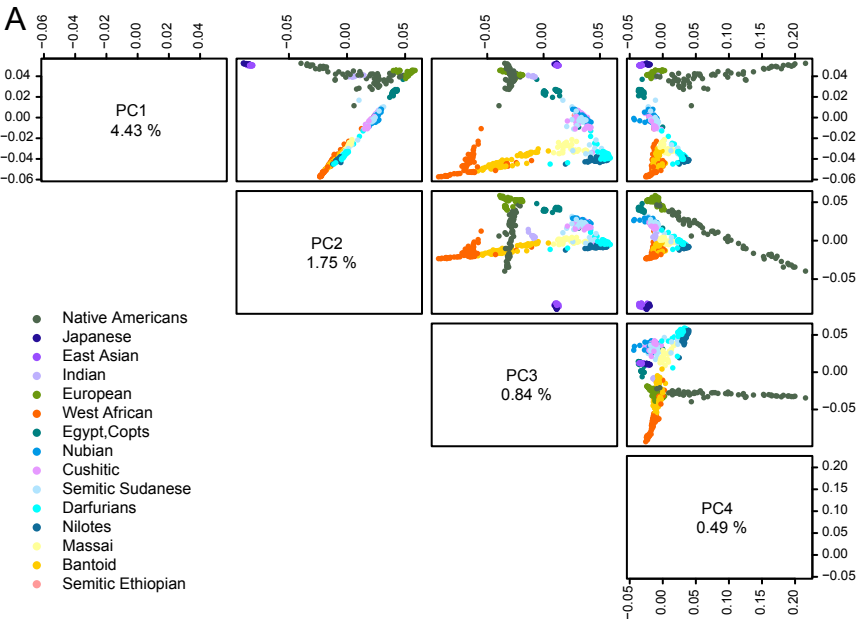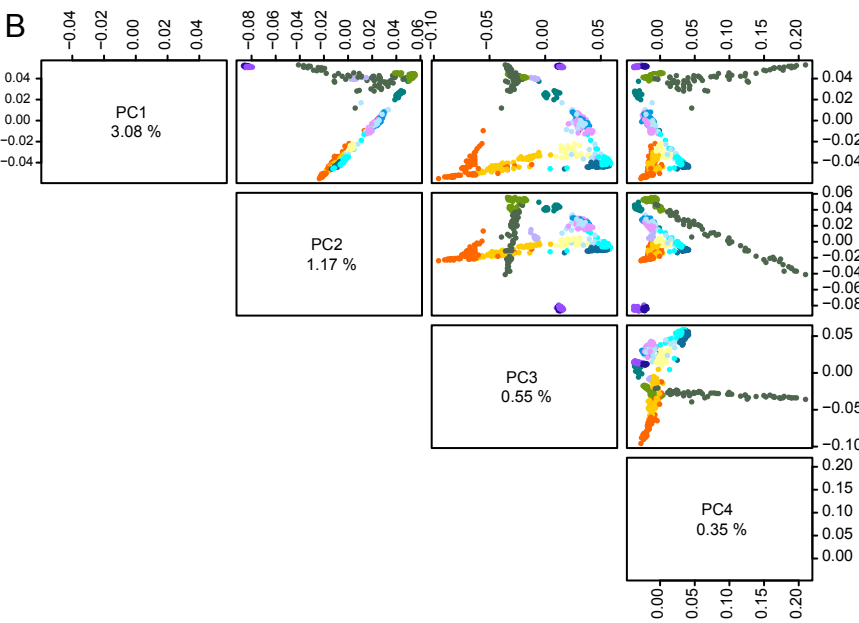

Supplement: S15 Fig — No outlier removal. (A) Diploid dataset. (B) Haploid dataset. (PDF) [file pgen.1006976.s016.pdf]

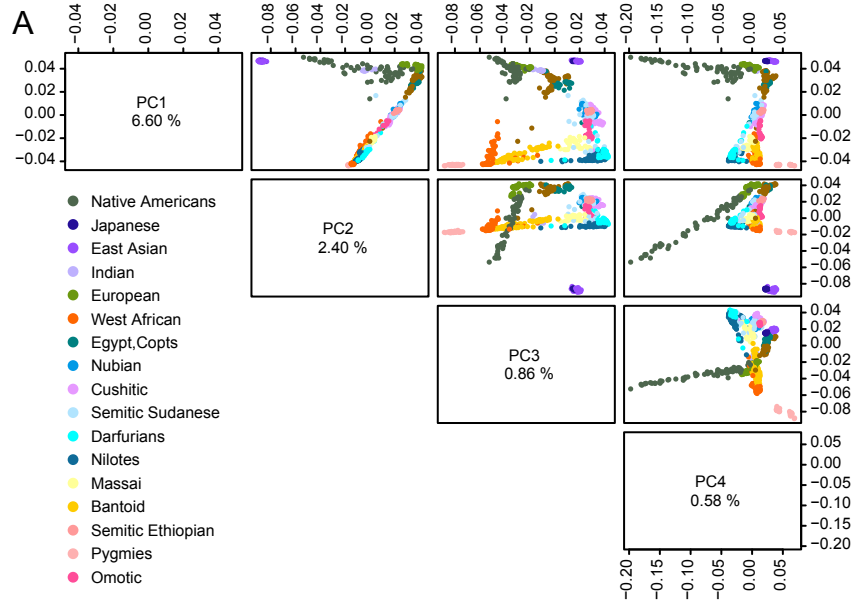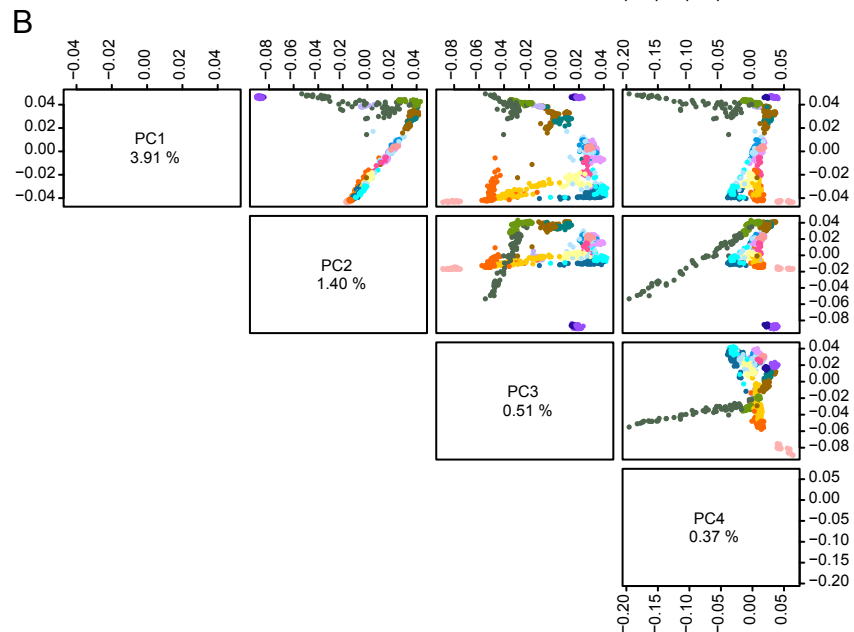

Supplement: S16 Fig — No outlier removal. (A) Diploid dataset. (B) Haploid dataset. (PDF) [file pgen.1006976.s017.pdf]

A

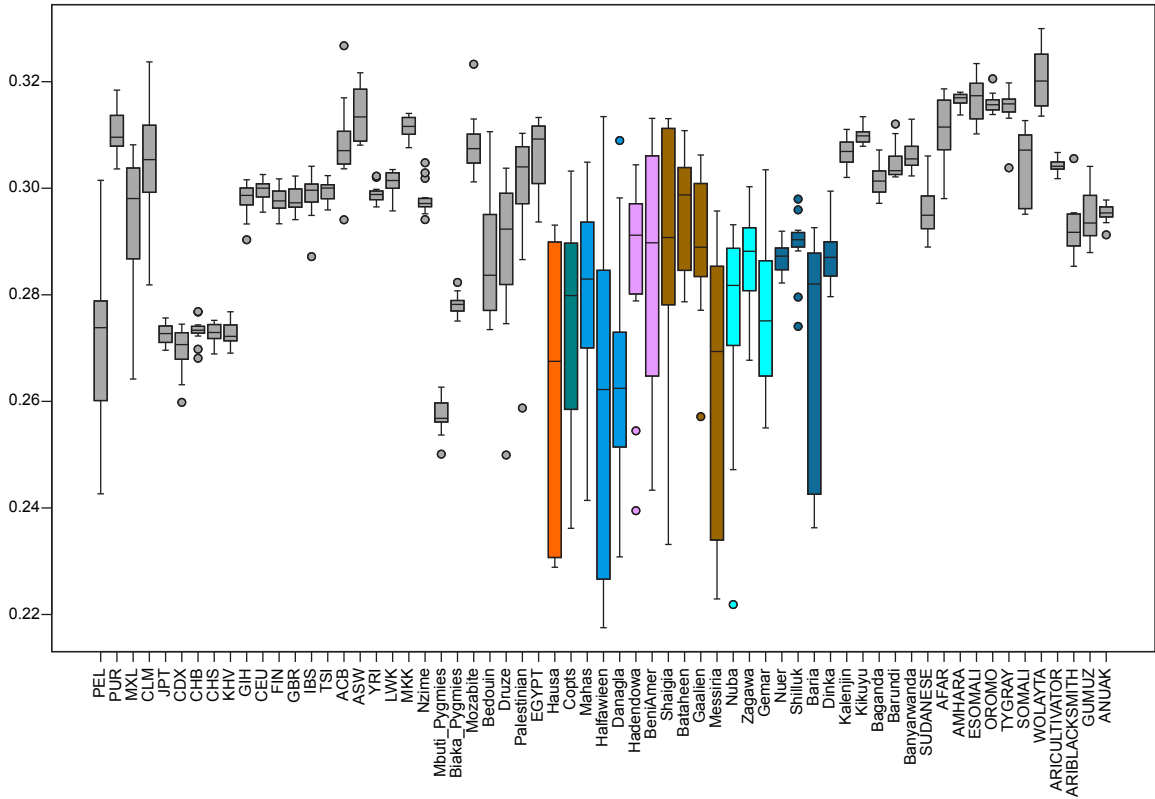

B

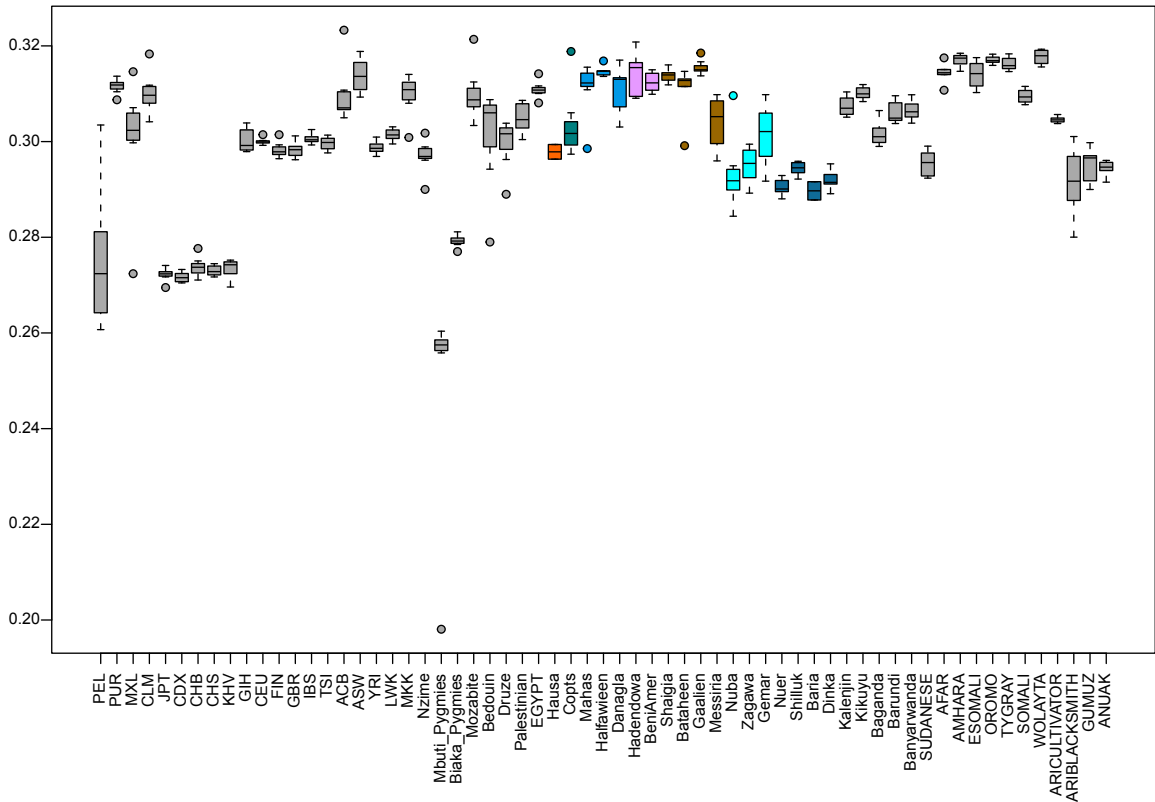

Supplement: S17 Fig — Heterozygosity is shown on the Y-axis. Sudanese populations are colored according to linguistic affiliation. Orange = Chadic, teal = Ancient Egyptian, blue = Nubian/Eastern Sudanic, pink = Cushitic, brown = Semitic, cyan = various Eastern Sudanic, and dark blue = Nilotic/Eastern Sudanic. (A) shows the heterozygosity for dat3. (B) shows the heterozygosity after the dataset was haploidized and chimeric individuals were created. This decreases the sample size by more than 50 percent. (PDF) [file pgen.1006976.s018.pdf]

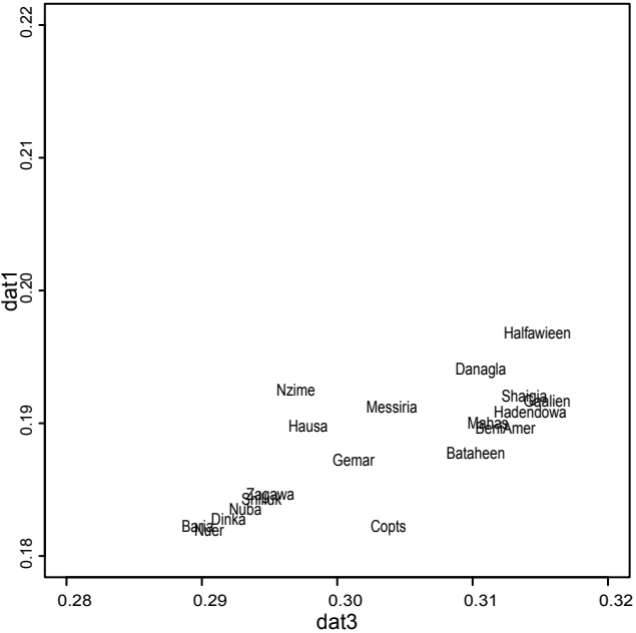

Supplement: S18 Fig — Average value of heterozygosity per population after removal of one outlier in the Nzime in dat1. (PDF) [file pgen.1006976.s019.pdf]

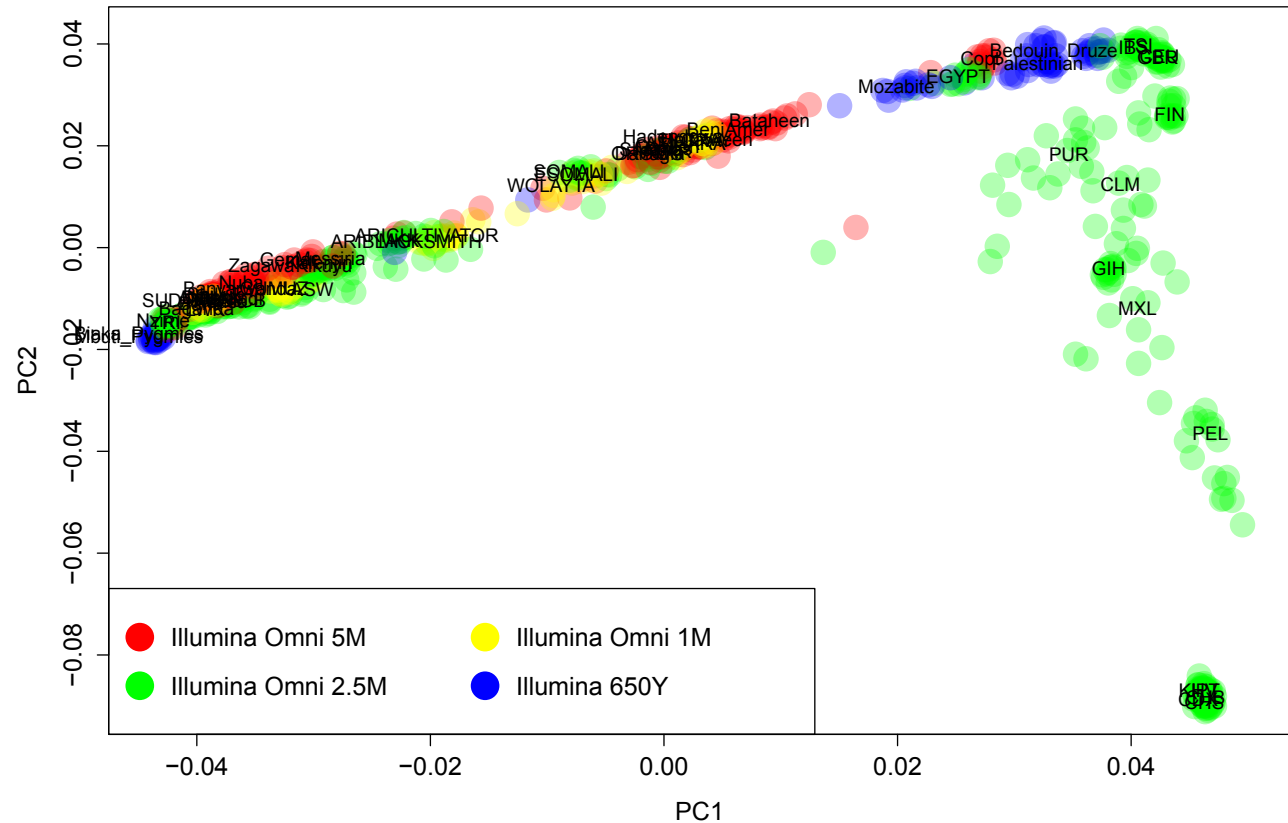

Supplement: S19 Fig — Samples are colored according to genotyping platform they were genotyped on. Population labels are displayed on the median of the individual values. (PDF) [file pgen.1006976.s020.pdf]

A

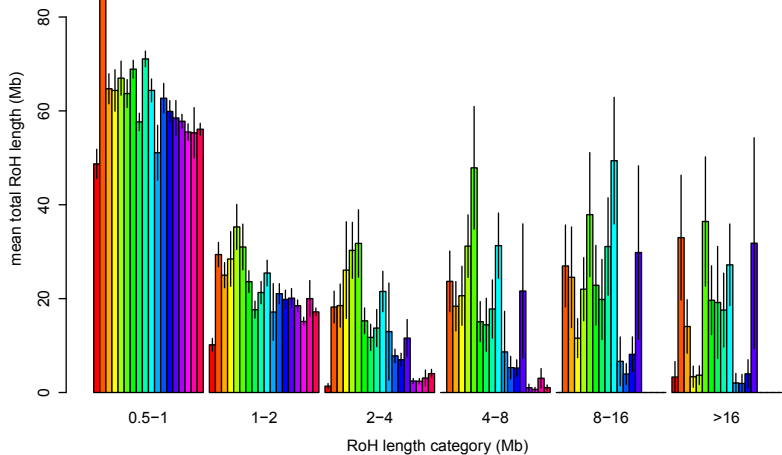

B

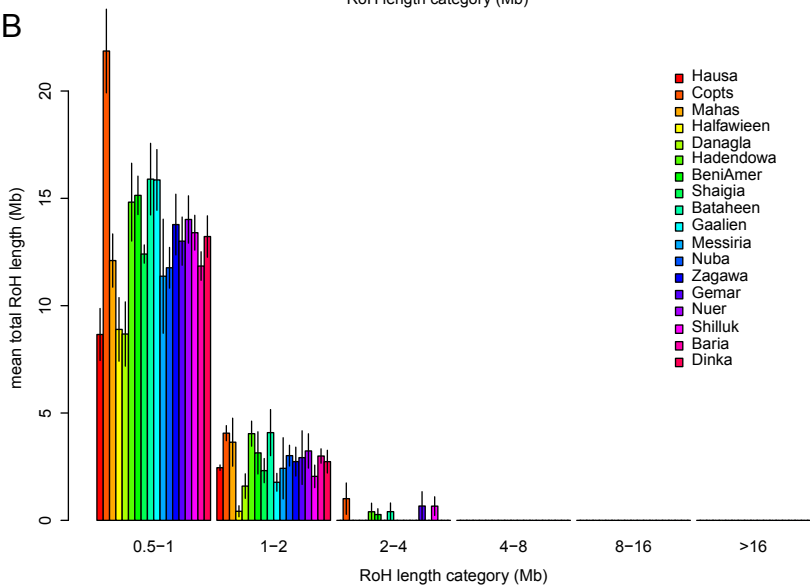

Supplement: S20 Fig — The average total length of the genome in runs of homozygosity in a number of length categories is plotted for each Sudanese population. Error bars represent one standard deviation. (A) Runs of homozygosity for the diploid dataset. (B) Runs of homozygosity of the chimeric unmerged dataset. Legend applies to both plots. (PDF) [file pgen.1006976.s021.pdf]

A

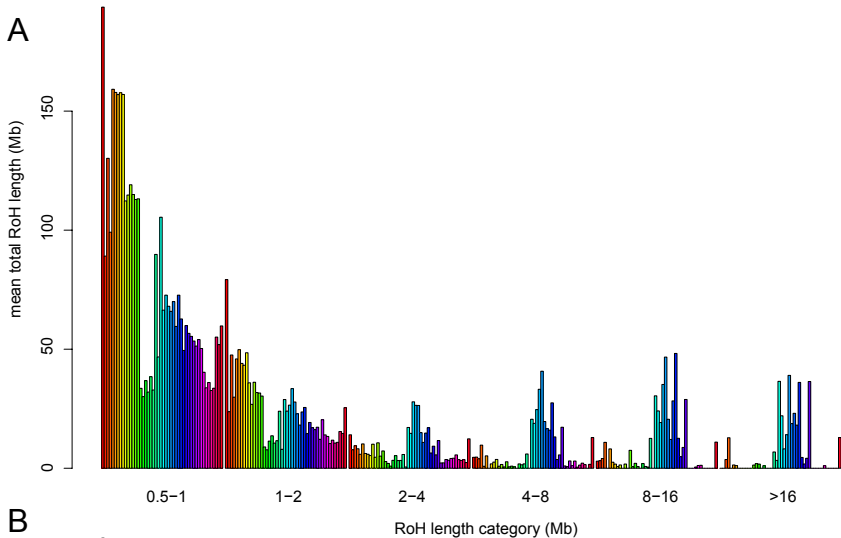

B

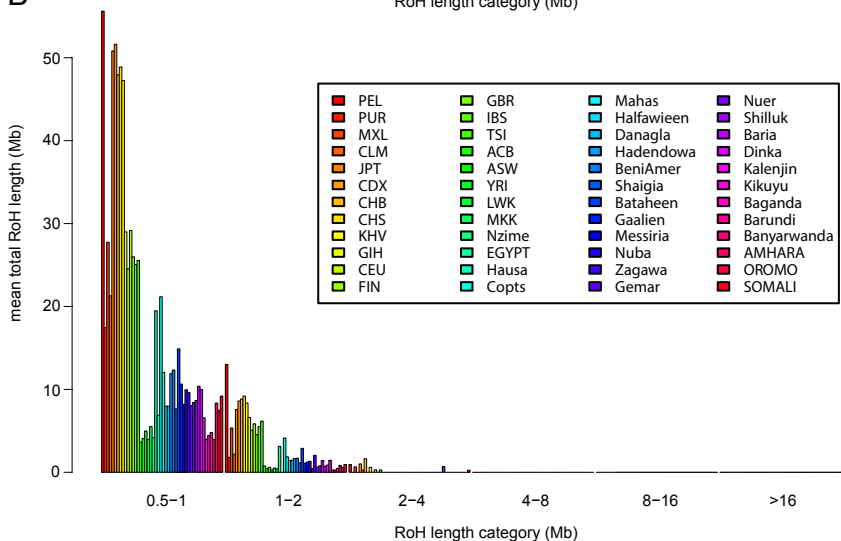

Supplement: S21 Fig — The average total length of the genome in runs of homozygosity in a number of length categories is plotted for each Sudanese population. Error bars represent one standard deviation. (A) Runs of homozygosity for the diploid dataset. (B) Runs of homozygosity of the chimeric unmerged dataset. Legend applies to both plots. (PDF) [file pgen.1006976.s022.pdf]

A

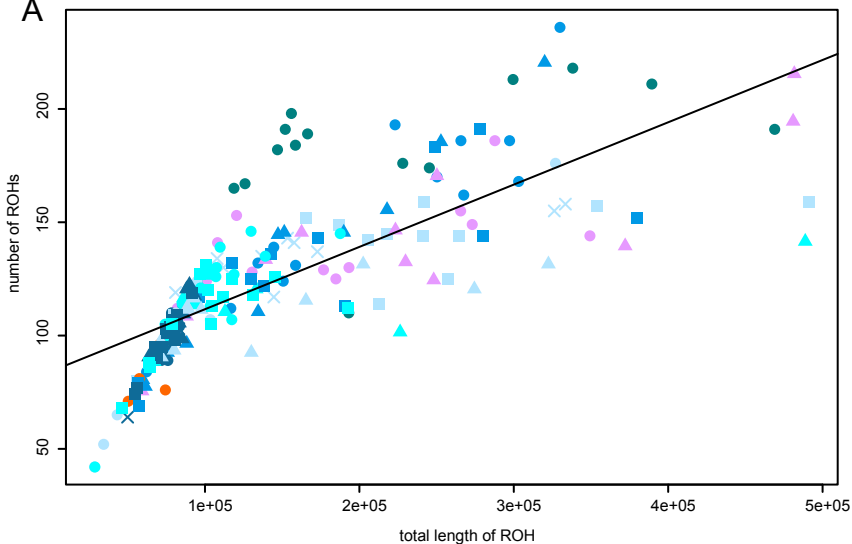

B

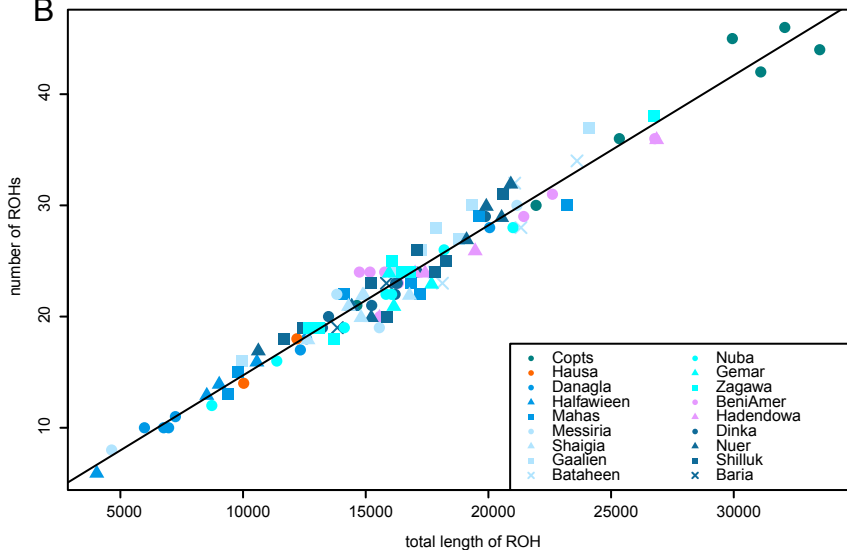

Supplement: S22 Fig — (A) Diploid dataset.(B) Chimeric dataset. (PDF) [file pgen.1006976.s023.pdf]

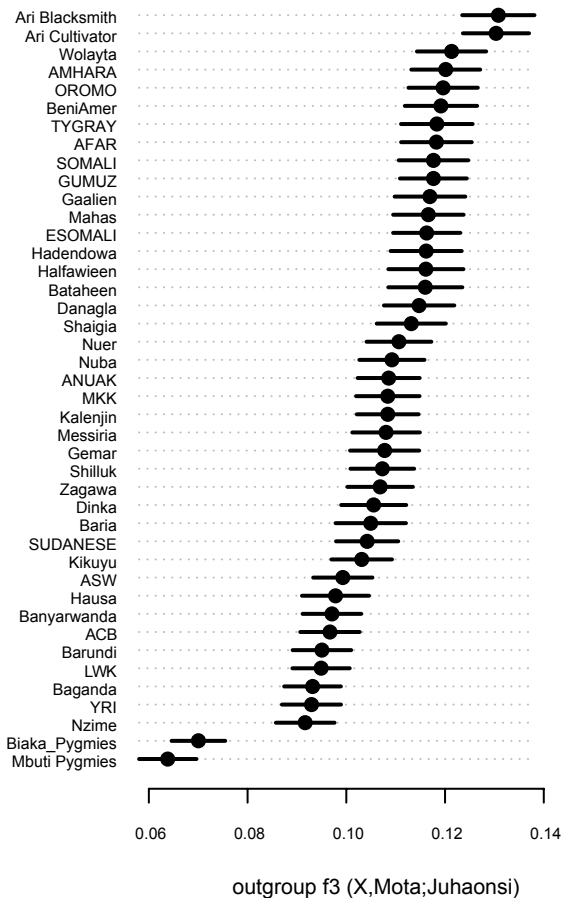

Supplement: S23 Fig — Measured shared drift of the populations on the Y axis with ancient Ethiopian individual. Lines indicate 2SE. (PDF) [file pgen.1006976.s024.pdf]

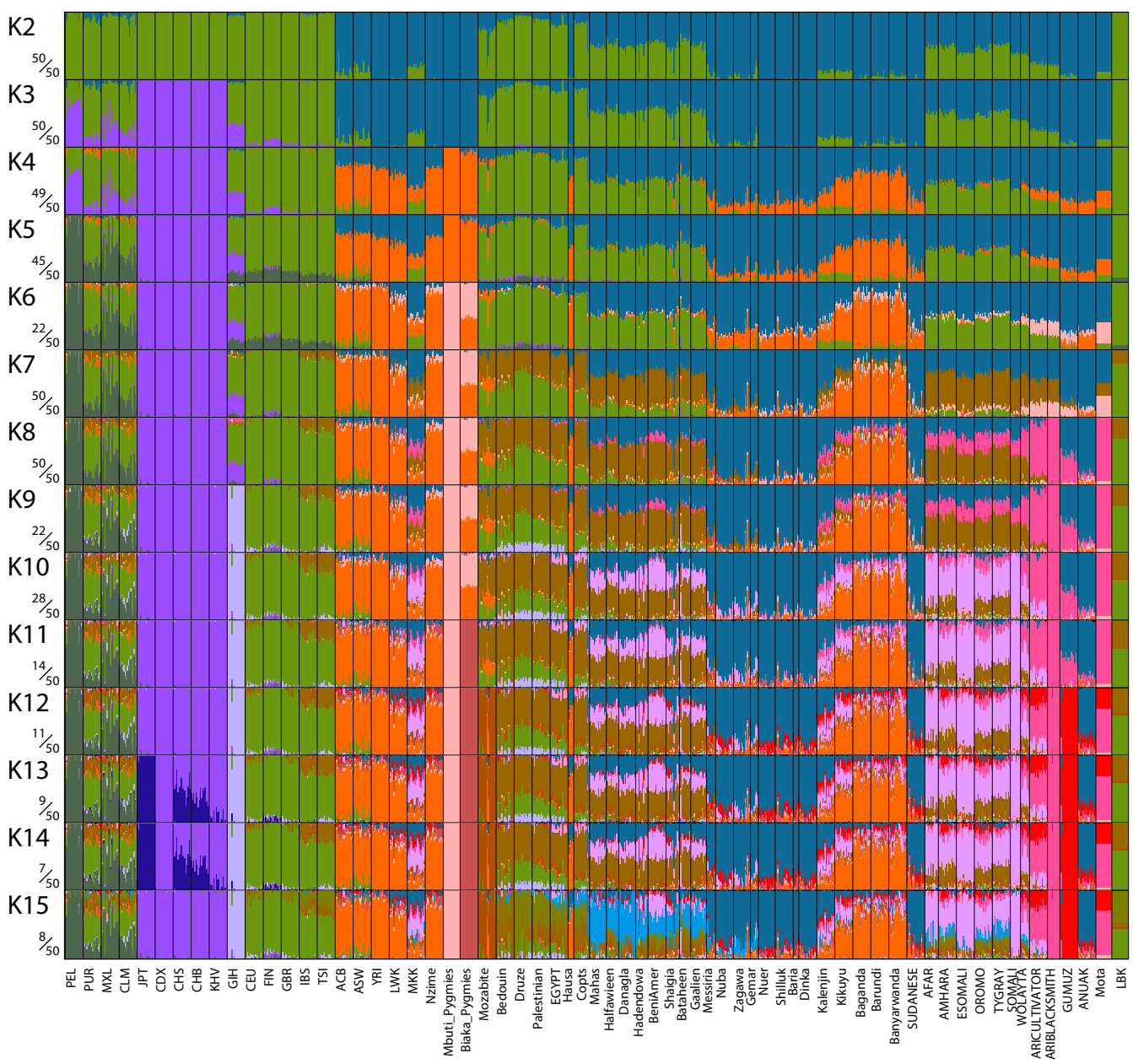

Supplement: S24 Fig — The cluster number can be found on the left along with the amount of iterations that support this cluster out of 50 (CLUMPP) [52, 53]. The ancient individuals are on the right. (PDF) [file pgen.1006976.s025.pdf]

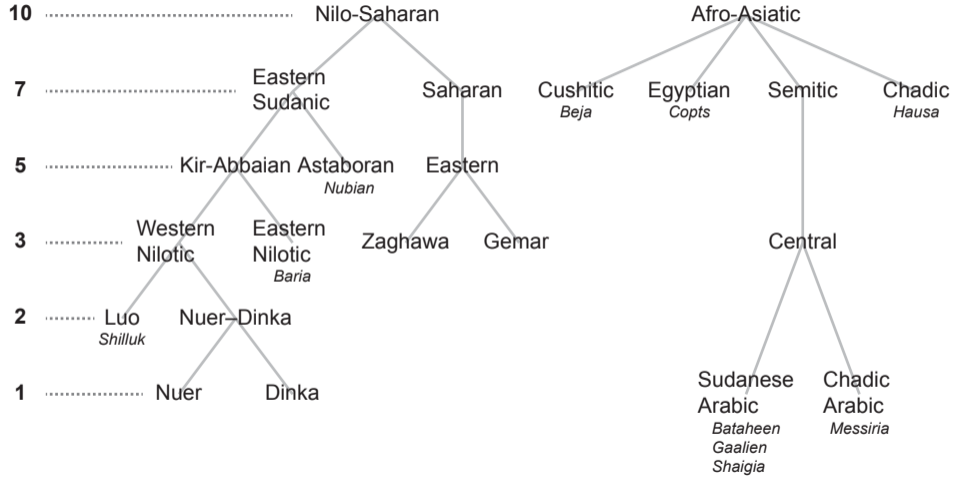

Supplement: S25 Fig — Classification according to Greenberg [54]. On the left are the distance values that are assigned at the first common node if the populations speak different languages. In italics are the populations that speak the language if the name of the language does not match the name of the population. (PDF) [file pgen.1006976.s026.pdf]

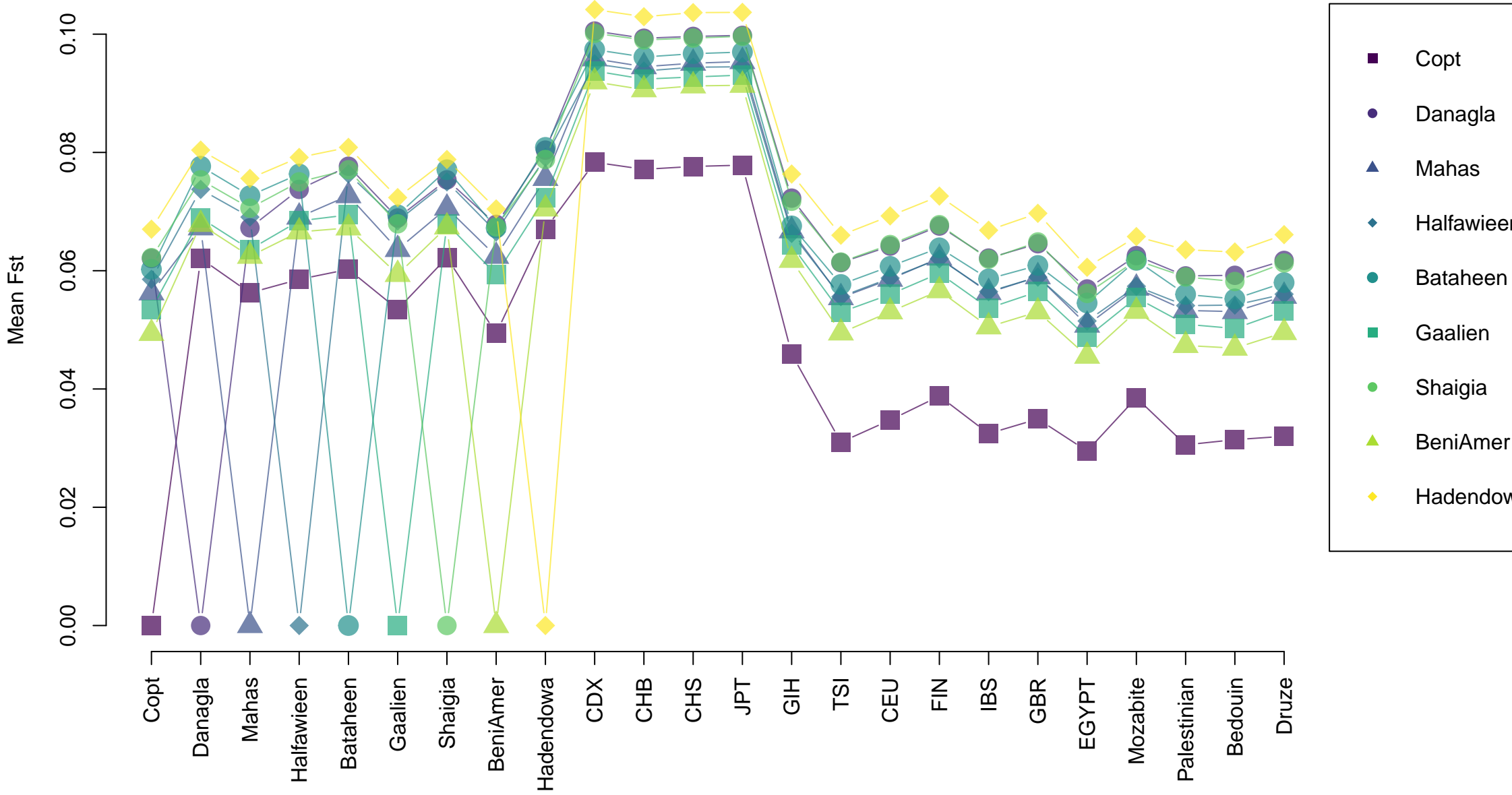

Supplement: S26 Fig — The allele frequencies were estimated by removing African allele frequencies (based on Nuer) to estimate which non-African population is closest to the donor population. (PDF) [file pgen.1006976.s027.pdf]

A

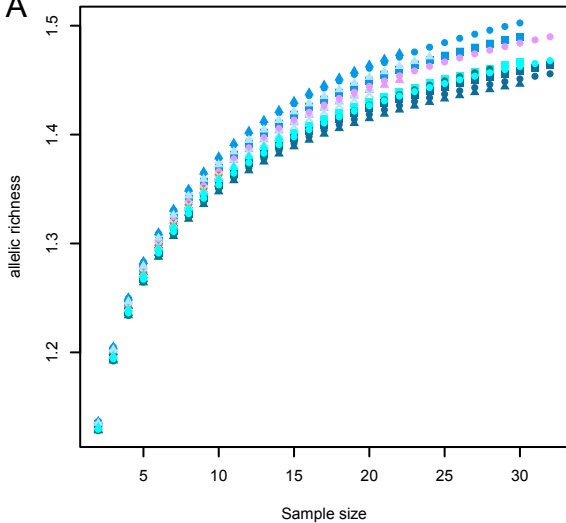

B

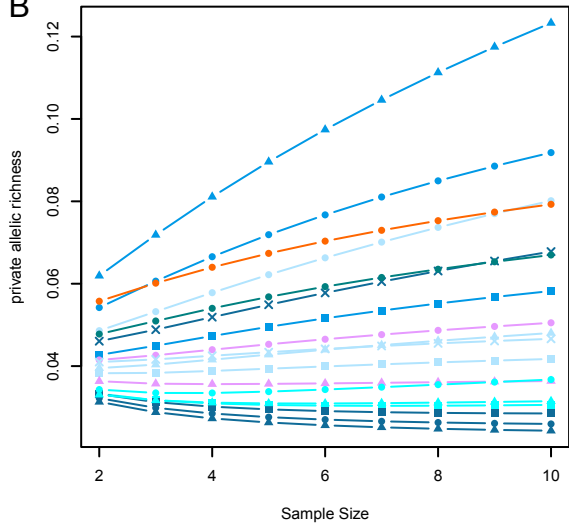

Supplement: S27 Fig — Five consecutive SNPs have been combined to create short haplotypes. A rank correlation test of the highest sample size (n = 10, Spearman) shows a high correlation (ρ = 0.9050568, p-value < 2.2e-16) with the ADZE result based on the SNPs. Removing the Copts from this increases the correlation slightly (ρ = 0.9240196). (PDF) [file pgen.1006976.s028.pdf]
