# Supplementary material for: Northeast African genomic variation shaped by the continuity of indigenous groups and Eurasian migrations
Source: PLoS Genet. 2017 Aug 24;13(8):e1006976. doi: 10.1371/journal.pgen.1006976 (PMC5587336; doi:10.1371/journal.pgen.1006976)
Supplement: S11 Fig — PC1 describes the variation between Africa—non-Africa. PC3 describes the African variation and differentiates the Pygmies, West Africans and East Africans. (PDF) [file pgen.1006976.s012.pdf]

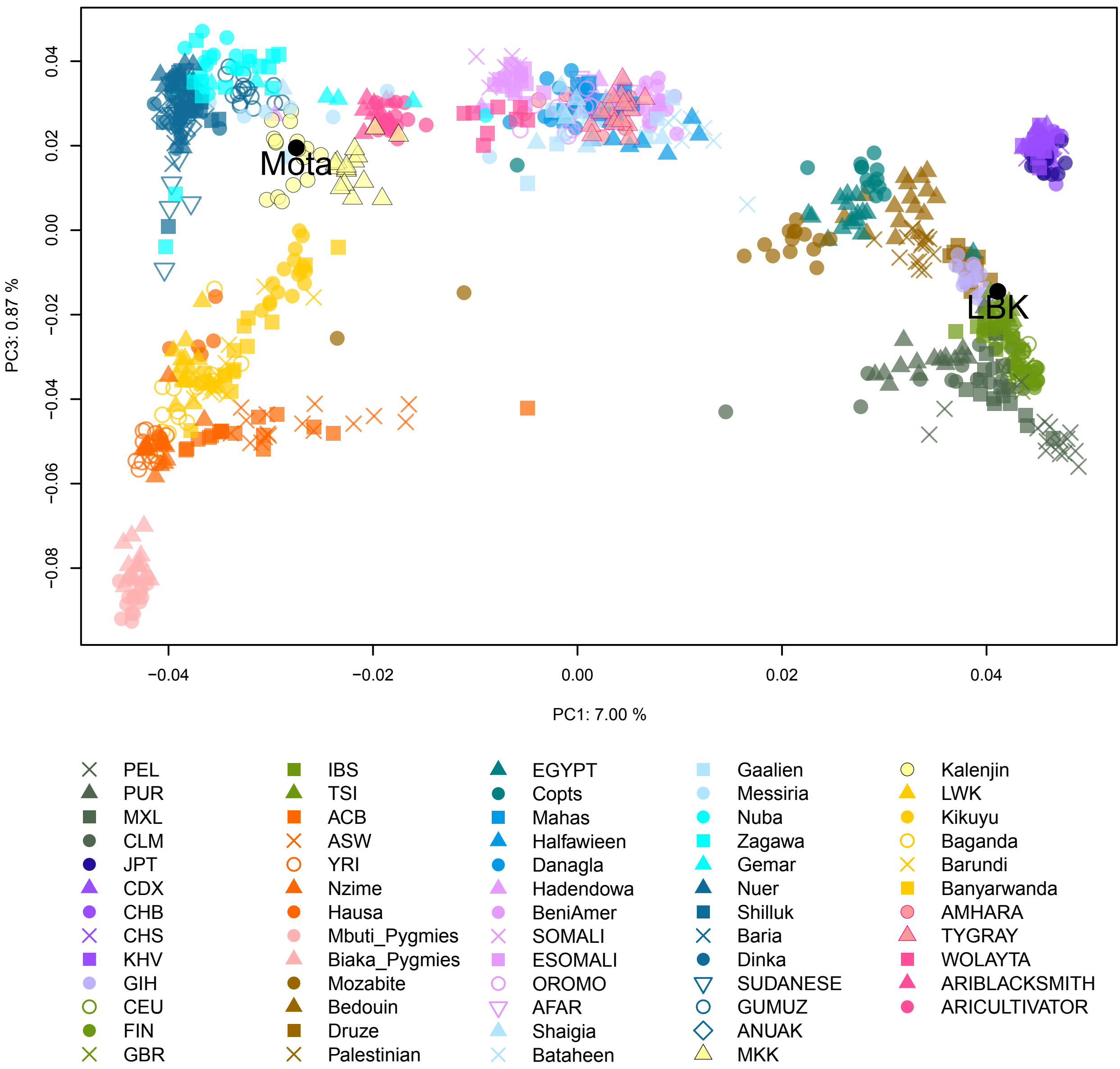

Mota
LBK
PC3: 0.87 %
PC1: 7.00 %
0.04
0.02
0.00
−0.02
−0.04
−0.06
−0.08
−0.04
−0.02
0.00
0.02
0.04
PEL
PUR
MXL
CLM
JPT
CDX
CHB
CHS
KHV
GIH
CEU
FIN
GBR
IBS
TSI
ACB
ASW
YRI
Nzime
Hausa
Mbuti_Pygmies
Biaka_Pygmies
Mozabite
Bedouin
Druze
Palestinian
EGYPT
Copts
Mahas
Halfawieen
Danagla
Hadendowa
BeniAmer
SOMALI
ESOMALI
OROMO
AFAR
Shaigia
Bataheen
Gaalien
Messiria
Nuba
Zagawa
Gemar
Nuer
Shilluk
Baria
Dinka
SUDANESE
GUMUZ
ANUAK
MKK
Kalenjin
LWK
Kikuyu
Baganda
Barundi
Banyarwanda
AMHARA
TYGRAY
WOLAYTA
ARIBLACKSMITH
ARICULTIVATOR
