# Supplementary material for: Northeast African genomic variation shaped by the continuity of indigenous groups and Eurasian migrations
Source: PLoS Genet. 2017 Aug 24;13(8):e1006976. doi: 10.1371/journal.pgen.1006976 (PMC5587336; doi:10.1371/journal.pgen.1006976)
Supplement: S1 Table — (PDF) [file pgen.1006976.s029.pdf]

Table S1: Overview of the datasets generated for further analysis

Dataset	Populations included	SNPs	Individuals	Additional Populations DatXa	SNPs DatXa	Individuals DatXa
**Dat1**	Sudanese<br>Nzime	3498433	247			
**Dat2**	3.5M SNP dataset<br>Ugandan and Ethiopian AGVP Populations<br>1000Genome Project<br>Egyptians	1391980	701	Mota<br>LBK<br>Juhoansi	140302	720
**Dat3**	1.4M SNP-dataset<br>Ethiopian populations<br>selected HGDP populations	220624	916	Mota<br>LBK<br>Juhoansi	20125	935
