# Supplementary material for: Northeast African genomic variation shaped by the continuity of indigenous groups and Eurasian migrations
Source: PLoS Genet. 2017 Aug 24;13(8):e1006976. doi: 10.1371/journal.pgen.1006976 (PMC5587336; doi:10.1371/journal.pgen.1006976)
Supplement: S2 Table — The table is sorted for the statistic value. (PDF) [file pgen.1006976.s030.pdf]

Table S2:lowest values of f3-statistics for targets tested against all possible source combinations. The table is sorted for the statistic value.

Target	***Source 1***	***Source 2***	***statistic***	***Z-score***
**Shaigia**	Nuer	TSI	-0.032156	-79.754
**Gaalien**	Nuer	TSI	-0.032016	-83.405
**Mahas**	Nuer	TSI	-0.029404	-71.75
**Danagla**	Nuer	TSI	-0.027881	-67.455
**Hadendowa**	Nuer	TSI	-0.027859	-59.965
**BeniAmer**	Nuer	TSI	-0.027326	-65.959
**Halfawieen**	Nuer	TSI	-0.026485	-63.727
**Bataheen**	Dinka	TSI	-0.024337	-51.907
**EGYPT**	YRI	TSI	-0.018259	-45.453
**Messiria**	Nuer	TSI	-0.017354	-34.477
**Copts**	Nuer	TSI	-0.013037	-30.249
**Gemar**	Nuer	GBR	-0.012241	-20.552
**Zagawa**	Nuer	IBS	-0.004192	-9.852
**Nuba**	Nuer	TSI	-0.003955	-10.488
**Shilluk**	Nuer	TSI	-0.002565	-7.951
**Hausa**	YRI	TSI	-0.001071	-1.825
**Dinka**	Nuer	YRI	-0.001038	-5.283
**Baria**				no negative statistic
**Nuer**				no negative statistic
