# Supplementary material for: Northeast African genomic variation shaped by the continuity of indigenous groups and Eurasian migrations
Source: PLoS Genet. 2017 Aug 24;13(8):e1006976. doi: 10.1371/journal.pgen.1006976 (PMC5587336; doi:10.1371/journal.pgen.1006976)
Supplement: S3 Table — The table shows the dates of admixture of two source populations to form a target populations. (PDF) [file pgen.1006976.s031.pdf]

Table S3: ALDER results. The table shows the dates of admixture of two source populations to form a target populations.

Target	***Source 1***	***Source 2***	***Result in generations***	***Standard Error***	***Z-Score***
**Mahas**	Nuer	TSI	33.4	3.35	9.98
**Danagla**	Nuer	TSI	32.38	3.01	10.76
**Halfawieen**	Nuer	TSI	18.61	3.47	5.37
**Bataheen**	Nuer	TSI	20.06	2	10.05
**Gaalien**	Nuer	TSI	19.65	2.16	9.1
**Shaigia**	Nuer	TSI	23.78	2.81	8.45
**Beni Amer**	Nuer	TSI	61.29	4.54	13.5
**Hadendowa**	Nuer	TSI	22.11	5.22	4.23
**Beni Amer**	Nuer	Esomali	62.62	9.96	5.73
**Gemar**	Sudanese (Pagani)	TSI	13.72	2.59	5.29
