# Supplementary material for: Northeast African genomic variation shaped by the continuity of indigenous groups and Eurasian migrations
Source: PLoS Genet. 2017 Aug 24;13(8):e1006976. doi: 10.1371/journal.pgen.1006976 (PMC5587336; doi:10.1371/journal.pgen.1006976)
Supplement: S4 Table — (PDF) [file pgen.1006976.s032.pdf]

Table S4: Rolloff results

Target	*Source 1*	*Source 2*	*Mean*	*SE*
Beni Amer	Somali	Nuer	74.562	6.972
Hadendowa	Somali	Nuer	58.696	9.904
Messiria	TSI	Nuer	13.46	2.276
