# Supplementary material for: Northeast African genomic variation shaped by the continuity of indigenous groups and Eurasian migrations
Source: PLoS Genet. 2017 Aug 24;13(8):e1006976. doi: 10.1371/journal.pgen.1006976 (PMC5587336; doi:10.1371/journal.pgen.1006976)
Supplement: S5 Table — (PDF) [file pgen.1006976.s033.pdf]

Table S5: Population sizes of the chimeric datasets.

Chimeric Dataset	*number of SNPs*	*number of Individuals*
**Dat1c**	3498433	115
**Dat2c**	1391980	346
**Dat3c**	220624	452
