# Supplementary material for: Northeast African genomic variation shaped by the continuity of indigenous groups and Eurasian migrations
Source: PLoS Genet. 2017 Aug 24;13(8):e1006976. doi: 10.1371/journal.pgen.1006976 (PMC5587336; doi:10.1371/journal.pgen.1006976)
Supplement: S6 Table — The table is sorted after the f3 column. (PDF) [file pgen.1006976.s034.pdf]

Table S6: Outgroup f3 comparing the shared drift of Messiria and Source 2. The table is sorted after the f3 column.

Source 1	***Source 2***	***Target***	***f3***	***std. err***	***Z***	***SNPs***
**Messiria**	Nuer	Juhoansi	0.144514	0.001921	75.211	76486
**Messiria**	Nuba	Juhoansi	0.149261	0.001949	76.598	77185
**Messiria**	Zagawa	Juhoansi	0.151188	0.001963	77.01	77231
**Messiria**	Gemar	Juhoansi	0.151664	0.002048	74.073	74806
**Messiria**	Shaigia	Juhoansi	0.153397	0.002075	73.936	76604
**Messiria**	Gaalien	Juhoansi	0.154031	0.002067	74.502	76884
**Messiria**	Bataheen	Juhoansi	0.154856	0.002106	73.545	75455
