# Supplementary material for: Northeast African genomic variation shaped by the continuity of indigenous groups and Eurasian migrations
Source: PLoS Genet. 2017 Aug 24;13(8):e1006976. doi: 10.1371/journal.pgen.1006976 (PMC5587336; doi:10.1371/journal.pgen.1006976)
Supplement: S7 Table — The five highest amplitudes of donor population combinations are shown in descending order. (PDF) [file pgen.1006976.s035.pdf]

Target	Number of admixture events	Sources	Time in generations ago	Z-score
Mahas	2	Baria;TSI<br>Baria;CEU<br>Sudanese;TSI<br>Nuer;TSI<br>Baria;GBR	58.3538 +/- 12.197	4.78402
		Anuak;IBS<br>CEU;Dinka<br>Anuak;CEU<br>CEU;Shilluk<br>IBS;Shilluk	12.1588 +/- 2.437	4.98922
Danagla	2	Baria;CEU<br>Baria;TSI<br>Baria;IBS<br>Baria;GBR<br>CEU;Nuer	54.4522 +/- 10.3436	5.26437
		Dinka;TSI<br>Shilluk;TSI<br>Dinka;GBR<br>GBR;Shilluk<br>CEU;Dinka	13.8556 +/- 3.83165	3.61608
Halfawieen	1	Baria;CEU<br>ANUAK;CEU<br>CEU;Dinka<br>Nuer;TSI<br>Baria;GBR	19.3144 +/- 3.81746	5.05949
Bataheen	1	Dinka;TSI<br>Baria;TSI<br>SUDANESE;TSI<br>Nuer;TSI<br>ANUAK;TSI	19.4232 +/- 2.04534	9.49631
Gaalien	1	Nuer;TSI<br>Baria;TSI<br>Dinka;TSI<br>CEU;Nuer<br>Baria;CEU	17.8237 +/- 2.23161	7.98691
Shaigia	1	Baria;TSI<br>Dinka;TSI<br>Nuer;TSI<br>Baria;CEU<br>ANUAK;TSI	22.8108 +/- 2.12977	10.7105
Beni Amer	2	Baria;CEU<br>Baria;TSI<br>CEU;Nuer<br>Druze;Nuer<br>CEU;Dinka	107.656 +/- 24.3726	4.41711
		ANUAK;GBR<br>GBR;Shilluk<br>ANUAK;TSI<br>GBR;Hausa	34.2468 +/- 9.63256	3.55532

Hadendowa	1	Dinka;GBR<br>Baria;GBR<br>Baria;Druze<br>Baria;CEU<br>Baria;TSI<br>CEU;Dinka	20.4075 +/- 5.02117	4.0643
Gemar*	1	Baria;TSI<br>Baria;CEU<br>SUDANESE;TSI<br>Baria;GBR<br>CEU;SUDANESE	13.364 +/- 2.9853	4.4766
Messiria	1	Baria;CEU<br>Baria;TSI<br>CEU;Dinka<br>Dinka;TSI<br>CEU;Nuer	7.24021 +/- 0.90907	7.96441
Zagawa*	1	ANUAK;TSI<br>ANUAK;GBR<br>ANUAK;IBS<br>Nuer;TSI<br>GBR;Nuer	20.7618 +/- 6.49427	3.19695
Nuba*	1	Biaka_Pygmies;CEU<br>Biaka_Pygmies;GBR<br>Baria;CEU<br>CEU;Nuer<br>CEU;SUDANESE	35.8164 +/- 11.6697	3.06917
Hausa*	1	GBR;YRI<br>Biaka_Pygmies;GBR<br>FIN;YRI<br>Bedouin;YRI<br>Palestinian;YRI	31.251 +/- 9.33749	3.34683
Nuer*	1	Baria;TSI<br>Baria;Hausa<br>Baria;YRI<br>CEU;Shilluk<br>CEU;YRI	19.1594 +/- 4.1226	4.64741
Dinka	0			
Baria*	1	Gemar;JPT<br>Biaka_Pygmies;CHS<br>CHS;Gemar<br>JPT;Zagawa<br>Mbuti_Pygmies;Shaigia	31.5847 +/- 9.95394	3.17308
Shilluk*	1	GBR;Nuer<br>Baria;GBR<br>Dinka;GBR<br>Baria;TSI<br>Nuer;TSI	7.43002 +/- 1.69951	4.37186
Copts	0			

*Alder could not find consistent decay rates concordant with these results
