# Supplementary material for: Northeast African genomic variation shaped by the continuity of indigenous groups and Eurasian migrations
Source: PLoS Genet. 2017 Aug 24;13(8):e1006976. doi: 10.1371/journal.pgen.1006976 (PMC5587336; doi:10.1371/journal.pgen.1006976)
Supplement: S7 Fig — (A) Weir and Cockerhams estimator of FST. (B) Wrights FST for pseudo-unadmixed allele frequencies. (PDF) [file pgen.1006976.s008.pdf]

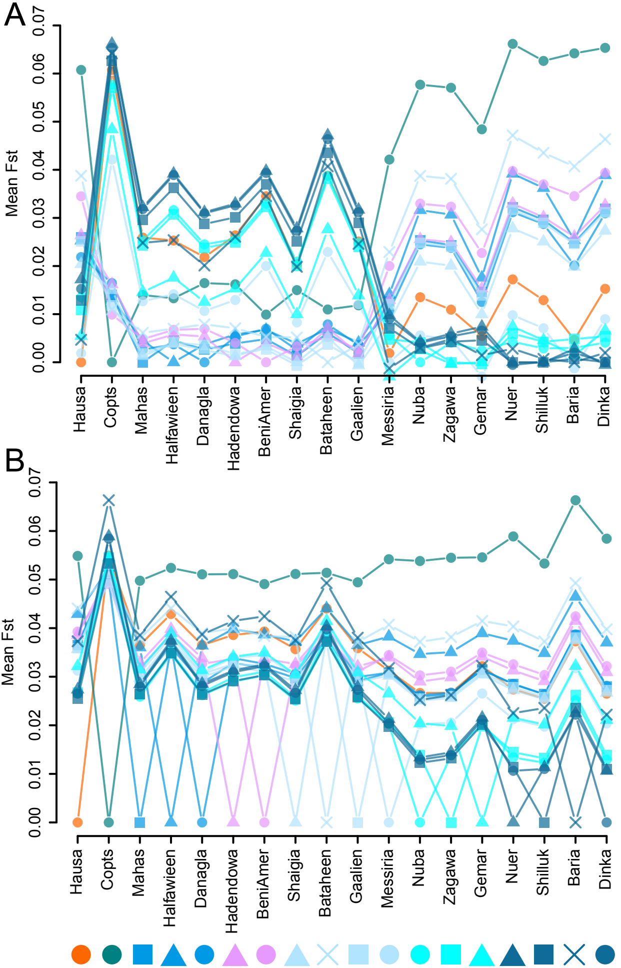
A
B
Mean Fst
0.00
0.01
0.02
0.03
0.04
0.05
0.06
0.07
Hausa
Copts
Mahas
Halfawieen
Danagla
Hadendowa
BeniAmer
Shaigia
Bataheen
Gaalien
Messiria
Nuba
Zagawa
Gemar
Nuer
Shilluk
Baria
Dinka
